# Eight Triplex-Binding Molecules from Four Chemical Classes Broadly Recognize the MALAT1 Triple Helix

**DOI:** 10.3390/molecules30214277

**Published:** 2025-11-03

**Authors:** Madeline M. Mousseau, Krishna M. Shivakumar, Jaesang Yoo, Jessica A. Brown

**Affiliations:** Department of Chemistry and Biochemistry, University of Notre Dame, Notre Dame, IN 46556, USA

**Keywords:** MALAT1, MENβ, small molecule, triple helix

## Abstract

RNA triple helices are relatively understudied, including their interactions with small molecules. In this study, we evaluated eight previously reported triplex-binding molecules (TBMs) for their functional effects on the premature and mature MALAT1 triple helix. Based on UV thermal denaturation experiments, the TBMs berberine, coralyne, sanguinarine, berenil, and neomycin selectively stabilize the Hoogsteen interface of the MALAT1 triple helix. Moreover, fisetin, luteolin, and quercetin were more sensitive to nucleotide composition, whereas berberine, coralyne, sanguinarine, and berenil were more sensitive to changes in the length of the major-groove triple helix. Most TBMs could not outcompete MALAT1 triple helix-binding proteins, except for neomycin. Surface plasmon resonance experiments demonstrated that berberine and sanguinarine display relatively quick association and dissociation binding profiles. Treating human colorectal carcinoma cells with each of the TBMs reduced MALAT1 levels by ~20–60%. This study demonstrates that TBMs broadly recognize the premature and mature MALAT1 triple helix but exhibit subtle sensitivities, suggesting that TBMs can be designed to selectively bind triple helices based on nucleotide composition, length, and structural context.

## 1. Introduction

**Figure 1:**
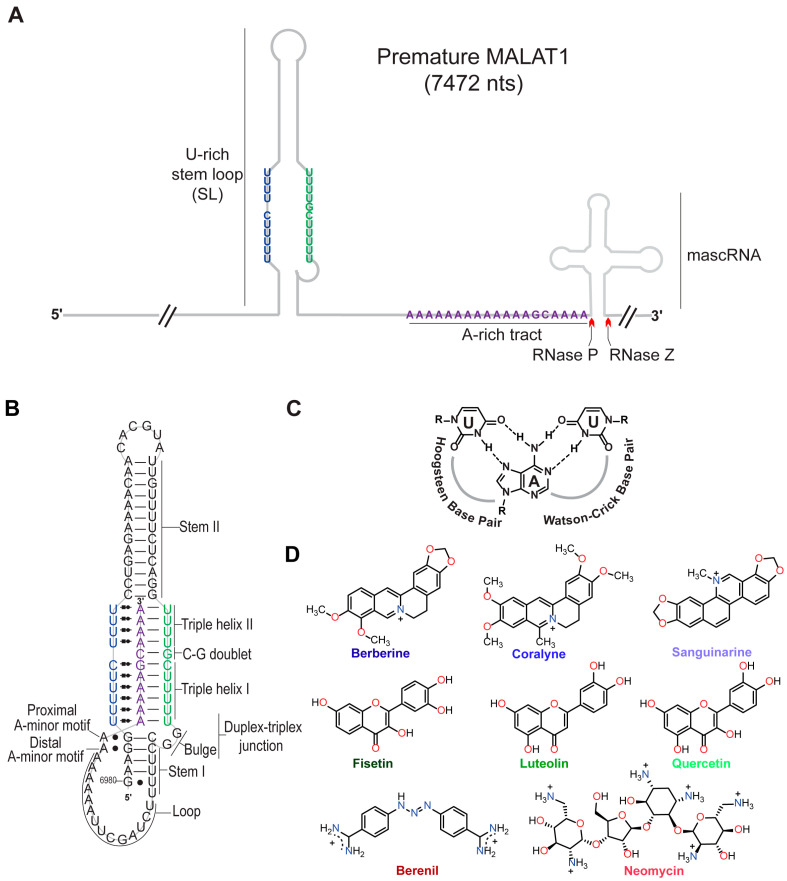
Structures of the MALAT1 triple helix and eight triplex-binding molecules (TBMs). (**A**) Cartoon schematic depicts the arrangement of the U-rich stem loop (SL), A-rich tract (A), and mascRNA at the 3′ end of premature human MALAT1, which is 7472 nts long. Red carats denote cleavage sites of RNases P and Z. Schematic is not drawn to scale. (**B**) Shown is a schematic depicting the secondary and tertiary structure of the mature MALAT1 triple helix. The Hoogsteen and Watson-Crick interactions are represented by Leontis-Westhof notation (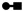) and a solid line (−), respectively. (**C**) Chemical structure of a U•A-U base triple is shown with Hoogsteen and Watson-Crick base pairing denoted by dashed lines. (**D**) Chemical structures of the eight TBMs examined in this study. The TBMs that are alkaloids are represented by a shade of blue: dark blue, blue, and light blue represent berberine, coralyne, and sanguinarine, respectively. TBMs that are flavonoids are represented by a shade of green: dark green, green, and light green represent fisetin, luteolin, and quercetin, respectively. The colors dark red and red represent berenil and neomycin, respectively.

Technological advancements have established the importance of long noncoding RNAs (lncRNAs) in various biological functions, and their dysregulation is associated with various diseases, including cancer [1,2]. One lncRNA that promotes metastasis of various cancer types is metastasis-associated lung adenocarcinoma transcript 1 (MALAT1); therefore, MALAT1 has emerged as a drug target, most notably its 3′ end [2,3,4,5,6]. At its 3′ end, MALAT1 contains a U-rich stem loop (SL), a genomically encoded A-rich tract (A), and a tRNA-like structure called MALAT1-associated small cytoplasmic RNA (mascRNA) (Figure 1A) [7,8,9,10]. pre-mascRNA is excised by RNases P and Z, generating a mature form of MALAT1 that forms a blunt-ended triple helix (SL+A) involving the U-rich SL and A-rich tract (Figure 1A,B) [7,10,11]. This predominantly U•A-U-rich triple helix (whereby Hoogsteen and Watson-Crick interactions are represented by a dot (•) and solid line (-), respectively) protects MALAT1 from degradation (Figure 1B,C) [8,9,11]. The MALAT1 triple helix has one confirmed protein-binding partner: the *N*^6^-methyladenosine writer protein methyltransferase-like protein 16 (METTL16) [12,13]. In addition to protein binding, several exogenous small molecules, which we henceforth refer to as triplex-binding molecules (TBMs), bind to the MALAT1 triple helix: diphenylfuran (DPF) derivatives [14,15], imidazole derivatives [16], berenil and its derivatives [17], 1,2,3-triazole derivatives [18], and conjugated and aromatic heterocyclic compounds [19,20,21,22]. Although these studies have focused on the mature MALAT1 triple helix, it may be more therapeutically advantageous if a small molecule interfered with the formation of the triple helix by targeting a premature state such as the U-rich SL that likely forms co-transcriptionally or the premature 3′ end consisting of the SL+A+masc (Figure 1A). Like the mature MALAT1 triple helix, several aromatic/heteroaromatic small molecules have targeted a predicted triple helix at the 3′ end of the multiple endocrine neoplasia-β (MENβ) lncRNA: aurintricarboxylic acid, mitoxantrone emodin, GW5074, mitoxantrone, and rottlerin [23]. These TBMs have been explored primarily for therapeutic purposes, although we still do not have a solid understanding of the basic chemical and physical properties that TBMs leverage to preferentially bind to triple-helical RNA structures.

Previously, small molecules belonging to the class of alkaloids (e.g., berberine [24,25,26], coralyne [25,27], and sanguinarine [24]), flavonoids (e.g., fisetin [28], luteolin [29], and quercetin [20,30]), triazene (berenil) [17,31], and aminoglycosides (neomycin) [32] (Figure 1D) were characterized for their interactions with generic nucleic acid structures: DNA/RNA double helices, DNA/RNA triple helices, and DNA G-quadruplexes. In general, most TBMs (alkaloids, flavonoids, and neomycin) stabilize the Hoogsteen face by ~3–20 °C and the Watson-Crick face by less than 5 °C [24,25,26,28,29,30,32]. Most of these TBMs are either dietary supplements (e.g., berberine, fisetin, luteolin, and quercetin) or FDA-approved drugs for veterinary (berenil) or human (neomycin) medical treatment. Except for berenil [17] and quercetin [20], the other six compounds (Figure 1D) have not been tested for their ability to engage with the MALAT1 triple helix, and none have considered the premature form of the MALAT1 triple helix (SL+A+masc).

Herein, we sought to study the selectivity, binding trends, and cellular effect of eight TBMs with the MALAT1 triple helix (Figure 1B,D). Our thermal melting studies confirm that most TBMs preferentially stabilize Hoogsteen interactions, similar to what has been observed previously for poly(U•A-U) or poly(T•A-T) triple helices [24,25,26,27,28,29,30,31,32]. When the nucleotide composition and length of the major-groove triple helix are altered, most alkaloids stabilize, whereas most flavonoids destabilize Hoogsteen interactions. Only neomycin can effectively prevent proteins from binding to the MALAT1 triple helix in the presence of HCT116 cell lysate. Five TBMs demonstrate fast binding interactions, although berberine and sanguinarine are the fastest. Steady-state levels of premature and mature MALAT1 in HCT116 cells were reduced in the presence of the flavonoids, berenil, and neomycin. Our study demonstrates that these first-generation TBMs interact with the MALAT1 triple helix and could potentially target both the mature and premature forms of the MALAT1 triple helix.

## 2. Results

### 2.1. Most TBMs Differentially Interact with the MALAT1 Triple Helix When Its Nucleotide Composition and Length Are Varied

**Figure 2:**
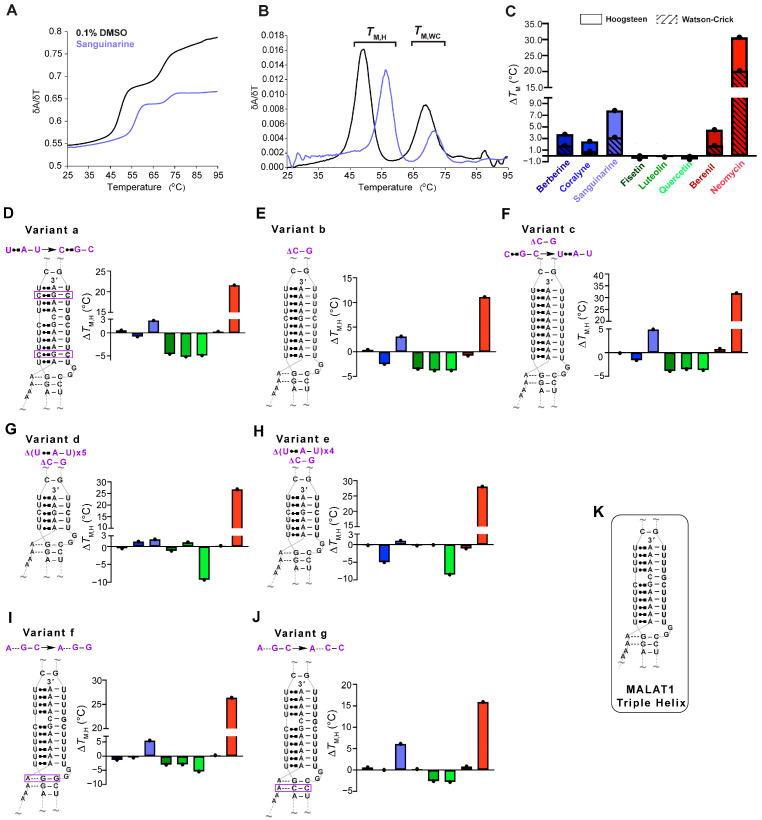
UV thermal denaturation results for the WT MALAT1 triple helix and MALAT1 variant RNAs in the absence and presence of TBMs. Plots of normalized (**A**) UV absorbance and (**B**) first derivative with respect to temperature for the MALAT1 triple helix in the absence (black line) and presence of sanguinarine (light blue). Bar plots show changes in the Hoogsteen (solid color) and the Watson-Crick (striped) melting temperatures for TBMs binding to (**C**) WT MALAT1, (**D**–**F**) variants with changes in nucleotide composition variants a–c, (**G**,**H**) shorter triple helices variants d and e, and (**I**,**J**) disrupted A-minor interactions are variants f and g. Bar colors correspond to the TBM as defined in Figure legend 1C. All bar plots are the average ∆*T*_M_ (n = 3), denoted by a single black dot. (**K**) A schematic is shown for the WT MALAT1 triple helix, focusing on the major-groove triple helix and A-minor motif. A tilde (~) denotes that peripheral RNA regions were present in the experiment but are not shown in the schematics for brevity. The Hoogsteen and Watson-Crick interactions are represented by Leontis-Westhof notation (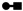) and a solid line (−), respectively. All RNAs used in this experiment are unimolecular, like the RNA shown in Figure 1B. All melting temperature values are compiled in Tables S1–S4. Raw and processed UV data are presented in File S1.

Our first objective was to determine if the eight TBMs preferentially target the major-groove triple helix of the MALAT1 triple helix as previously observed for poly(U•A-U) and poly(T•A-T) triple helices [24,25,26,28,29,30,31,32]. We employed UV thermal denaturation assays. As reported previously for the MALAT1 triple helix [9,14,33,34], its UV melting curve was biphasic, whereby the first derivative plot shows two distinct peaks: melting of primarily Hoogsteen interactions (*T*_M,H_), and likely the stem I and loop region, at 49.6 ± 0.4 °C and melting of the Watson-Crick interactions (*T*_M,WC_), particularly stem II, at 69 °C (Figure 1B and Figure 2A,B, Table 1). Because previous studies reported self-association of select TBMs, we next determined the UV melting profiles for each TBM at 10 µM in the absence of RNA (Figure S1A–C, File S1) [35,36,37,38,39]. Although peaks were observed, most were 0.3 to 6% of the peak heights that we determined for the MALAT1 triple helix in the absence of TBMs (Figure 1A,B and Figure S1D–F), except for quercetin having a shoulder peak at ~35 °C, which could be self-association of quercetin (Figure 1D and Figure S1D–F). These control assays establish that a positive or negative thermal shift (∆*T*_M_), respectively, indicates a net stabilization or destabilization of RNA structure in the presence of the TBM, likely induced by changes in non-covalent interactions between apo- and the TBM-bound MALAT1 triple helix states. All TBMs had a greater impact on ∆*T*_M,H_ than ∆*T*_M,WC_; therefore, we focused on ∆*T*_M,H_ (Figure 2C, Table 1). We note that ∆*T*_M_ values greater than 2.0 °C are considered significant because they are generally two times the standard deviation. The greatest stabilization was observed for neomycin with a ∆*T*_M,H_ of 30.7 °C (Figure 2C, Table 1). All alkaloids and berenil have ∆*T*_M,H_ values of ~2.5 to 8 °C (Figure 2C, Table 1). In contrast, the flavonoids mildly destabilize the MALAT1 triple helix with ∆*T*_M,H_ values of −0.2 °C to −0.6 °C (Figure 2C, Table 1). We also examined the flavonoids at 2% DMSO because that was previously used for quercetin [20]. There was no effect (i.e., ∆*T*_M,H_ = 0.1 °C) on the MALAT1 triple helix stability at 2% DMSO (Figure S2). Overall, the ∆*T*_M,H_ values ranged from approximately ~3–31 °C and were 2- to 3-fold greater than ∆*T*_M,WC_ values. Thus, TBMs exert a greater thermal effect on interactions that stabilize the first melting transition, suggesting that TBMs likely alter Hoogsteen base pairs of the major-groove triple helix and/or base pairs in the stem I/loop region. To evaluate likely binding sites, we used AlphaFold 3 to predict the MALAT1 triple helix-TBM complexes using the MALAT1 triple helix crystal structure (PDB ID: 4plx) [11,40]. For all TBMs, the most favorable binding sites were in the regions that contribute to *T*_M,H_ (i.e., duplex-triplex junction, triple helix I, and triple helix II) and not *T*_M,WC_ (Figure 1B and Figure S3).

Next, we were interested in determining whether TBM binding to the wild type (WT) MALAT1 triple helix is sensitive to nucleotide composition and/or the length of the major-groove triple helix. To maintain biological relevance, the full-length MALAT1 triple helix (Figure 1B), including the peripheral regions stem I, II, and loops, was used, akin to other similar studies [14,17,20,23,41]. We first assessed nucleotide composition by replacing two U•A-U base triples with isosteric C^+^•G-C base triples (variant a, Figure 2D), deleting the C-G doublet (variant b, Figure 2E), and deleting the C-G doublet as well as replacing the C^+^•G-C base triple with U•A-U (variant c, Figure 2F) so that the triple helix is ten U•A-U base triples. For variants a–c, there were a few notable differences for the ∆*T*_M_,_H_ values compared to WT: coralyne and all three flavonoids destabilized the MALAT1 variants by 0.8 to 5.2 °C, while berberine and berenil had almost no effect on ∆*T*_M,H_ (Figure 2C–F, Tables S1 and S2). In general, coralyne and the flavonoids are sensitive to changes in nucleotide composition of the MALAT1 triple helix.

In addition to nucleotide composition, we also probed the length of the major-groove triple helix by shortening it to five (variant d) and six (variant e) base triples because most structurally validated naturally occurring RNA triple helices have only three to five base triples (Figure 2G,H) [42]. Most TBMs had no significant impact on ∆*T*_M,H_ with two exceptions. One, quercetin induced a destabilization of ~9 °C and two, coralyne destabilized a six-base triple-long major-groove triple helix but not five (Figure 2G,H, Tables S3 and S4). To provide structural insights into why coralyne was sensitive to a one-base triple difference, we used FpocketR, a software tool that predicts binding pockets on a provided RNA target [43,44]. FpocketR showed different binding pockets along the duplex-triplex junction for both variants d and e; however, the pocket for variant e extends into stem I more than variant d (Figure S4), suggesting that coralyne binding may destabilize that part of the MALAT1 triple helix. Additionally, most TBMs were predicted to bind in triplex I or the duplex-triplex junctions (Figure 1B and Figure S3). These results suggest that TBMs have the ability to differentially recognize structural features of major-groove triple helices.

Lastly, we examined variants f and g (Figure 2I,J) that, respectively, disrupt the proximal and distal A•G-C minor-groove base triples, a location that might represent favorable binding pockets as previously identified for DPFp8 (Figure S5) [15] and by our computationally predicted MALAT1 triple helix-TBM complexes (Figure S3). For both variants, f and g, the alkaloids had no major effect except for sanguinarine, which increased *T*_M,H_ by about 6 °C (Figure 2I,J). The flavonoids destabilized variants f and g by about 2.5 to 5.5 °C, except fisetin had no effect on variant g (Figure 2I,J, Table S4). These results suggest that fisetin may bind near the A-minor base triples, which is the predicted binding site in our MALAT1 triple helix-fisetin complex (Figure 2I,J, and Figure S3). In summary, our results show that flavonoids are more sensitive to nucleotide composition, alkaloids are more sensitive to changes in nucleotide length, berenil selectively stabilized only the WT MALAT1 triple helix, and neomycin hyperstabilized all MALAT1 triple helices. These results suggest that these TBMs have subtle tunability with respect to certain structural features.

### 2.2. Only Neomycin Prevents the Formation of a MALAT1 Triple Helix RNP Complex

**Figure 3:**
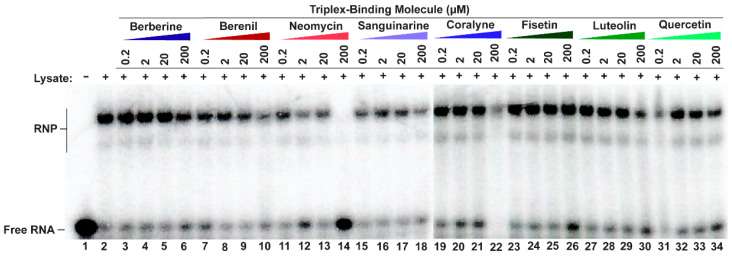
Competitive EMSA showing most TBMs cannot disrupt RNP complex formation. A 5′-[^32^P]-radiolabeled MALAT1 triple helix was incubated in the presence of HCT116 cell lysate and increasing amounts of a competitor TBM. Please note that there are two native gel-shift images because not all samples could be loaded onto a single gel; the divide occurs between lanes 18 and 19.

We next sought to probe each TBM’s ability to selectively target the MALAT1 triple helix in the presence of its protein-binding partners, such as METTL16 [12]. Although the exact binding interaction is unknown for the METTL16•MALAT1 triple helix complex, one would expect that the TBMs could prevent binding of METTL16 if they preferentially bind to a similar region of the major-groove triple helix region as does METTL16 [12]. Therefore, a competitive electrophoretic mobility shift assay (EMSA) was performed with increasing amounts of TBMs (0.2, 2, 20, and 200 µM) in the presence of whole-cell lysate extracted from HCT116 cells, a human colorectal carcinoma cell line. As observed previously using HEK293 cell lysate, ribonucleoprotein (RNP) complex formation also occurred in the presence of HCT116 cell lysate (Figure 3, lane 2) [12]. All TBMs, except for 200 µM neomycin (Figure 3, lane 14), did not reduce RNP formation (Figure 3, lanes 3–13, 15–34). This result is not too surprising considering these TBMs are known to bind multiple cellular targets, such as various proteins [45,46,47], ribosomes [48], microtubules [49,50], and G-quadruplexes [51,52,53,54] (Table S5). Therefore, it is possible that the TBMs are binding to these other cellular components instead. These TBMs cannot outcompete MALAT1 triple helix-binding proteins, such as METTL16 [12], suggesting either off-target binding by the TBMs or non-overlapping binding sites.

### 2.3. Select TBMs Interact with RNAs Mimicking the Premature MALAT1 Triple Helix States

**Figure 4:**
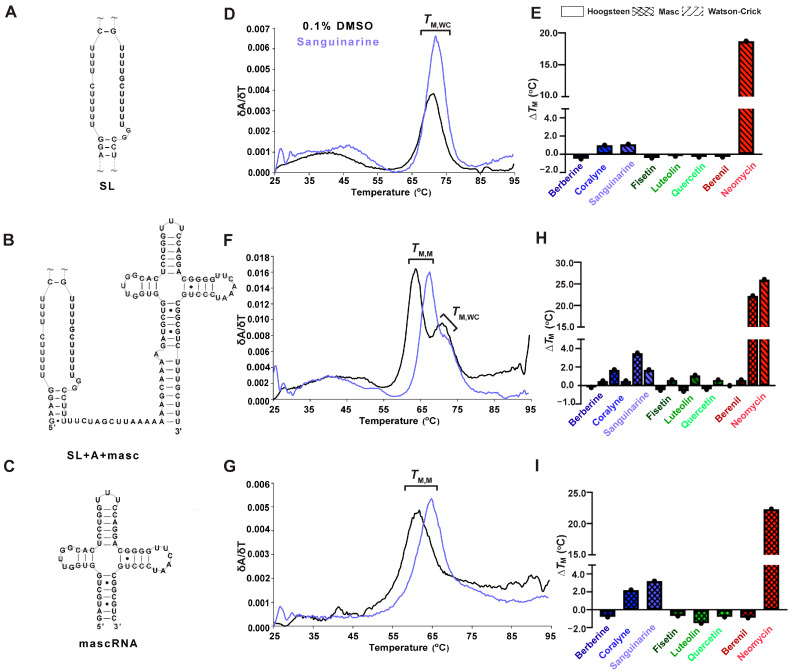
UV thermal melting results of premature MALAT1 RNAs in the absence and presence of TBMs. For each RNA, a schematic denoting the MALAT1 (**A**) SL, (**B**) SL+A+mascRNA, and (**C**) mascRNA is on the left; (**D**,**F**,**G**) first derivative versus temperature plot is in the center; and (**E**,**H**,**I**) a bar plot showing change in melting temperatures (Δ*T*_M_) is shown for each TBM binding to the MALAT1 RNAs. All bar plots display the average ∆*T*_M_ (n = 3), denoted by a single black dot. A tilde (~) denotes that peripheral RNA regions were present in the experiment but are not shown in the schematic for brevity. All RNAs used in this experiment are unimolecular, like the RNA shown in Figure 1B. All melting temperature values are compiled in Tables S7 and S8, and first derivative plots for all TBMs are presented in Figure S7. Raw and processed UV data are presented in File S1.

Because RNA folding occurs co-transcriptionally and the 3′ end of MALAT1 likely undergoes processing prior to formation of the mature MALAT1 triple helix (SL+A) [7,10,55], we were interested in determining whether TBMs could also target the premature MALAT1 RNAs (i.e., MALAT1 SL and MALAT1 SL+A-rich tract+mascRNA (SL+A+masc)) as well as mascRNA to monitor effects due to that unique structure [10] (Figure 4A–C, Table S6). The SL, which cannot form a triple helix but still contains structural motifs such as single mismatches and bulged loops like SL+A, exhibits a single-peak melting profile, i.e., only the *T*_M,WC_ peak at 71.2 ± 0.3 °C (Figure 4A,D, Table S7). In general, the ∆*T*_M,WC_ values were similar for both SL and SL+A in the presence of TBMs, except berberine, sanguinarine, and berenil exhibited about a 2 °C destabilization relative to SL+A (Table 1 and Table S8). This result suggests that the TBMs bind to a major-groove triple helix as opposed to an RNA lacking base triples (Figure 2C and Figure 4E, Tables S7 and S8).

MALAT1 SL+A+masc results in two distinct melting transitions at 64.0 ± 0.3 and 70.1 ± 0.5 °C (Figure 4F, Table S7). The peak at 70.1 ± 0.5 °C is assigned to *T*_M,WC_ because it is similar to *T*_M,WC_ peaks observed for SL and SL+A RNAs (Figure 2B and Figure 4D,E, Table 1 and Table S7). We hypothesized that the other peak represents the melting of mascRNA, and indeed the melting temperature for pre-mascRNA (64.0 ± 0.1 °C, Figure S6) and mascRNA (61.6 ± 0.1 °C, Figure 4G, Table S7) corresponds to 64.0 ± 0.3 °C for SL+A+masc (Figure 4F, Table S7). Therefore, we assigned that peak as *T*_M,M_, where “M” represents mascRNA (Figure 4F,G). Please note that both SL and SL+A+masc have a broad transition present at ~48.5 ± 0.9 °C for no TBM added (Figure 4D,F). This peak may represent non-canonical U•U base pairs in the U-rich internal loop [56]. These short, broad peaks are interpreted as low-confidence because (i) their peak height is only 15% of WT peak heights and (ii) they are not reproducible in all replicates (Figure S7, Table S7 and File S1). We report the values in Table S7 but do not discuss them due to their uncertainty compared to *T*_M,M_ and *T*_M,WC_.

The ∆*T*_M,M_ values for SL+A+masc and mascRNA are mostly similar. Mild destabilization (−0.2 to −1.5 °C) is observed for berberine, all flavonoids, and berenil, whereas coralyne, sanguinarine, and neomycin stabilized RNAs by 1.7 to 23 °C (Figure 4H,I, Table S8). The ∆*T*_M,WC_ values of SL+A+masc are comparable to SL+A and SL, except for luteolin, which stabilizes 1.1 °C (Figure 4E,H, Table S8). These results suggest that the flavonoids do not significantly stabilize or destabilize premature or mature MALAT1 RNAs. Additionally, berberine and berenil could be promising therapeutics for targeting mature MALAT1, as they alter the thermal stability for the SL+A at a higher magnitude compared to SL, SL+A+masc, and mascRNA.

### 2.4. Most TBMs Rapidly Associate with the Premature and Mature MALAT1 Triple Helix

**Figure 5:**
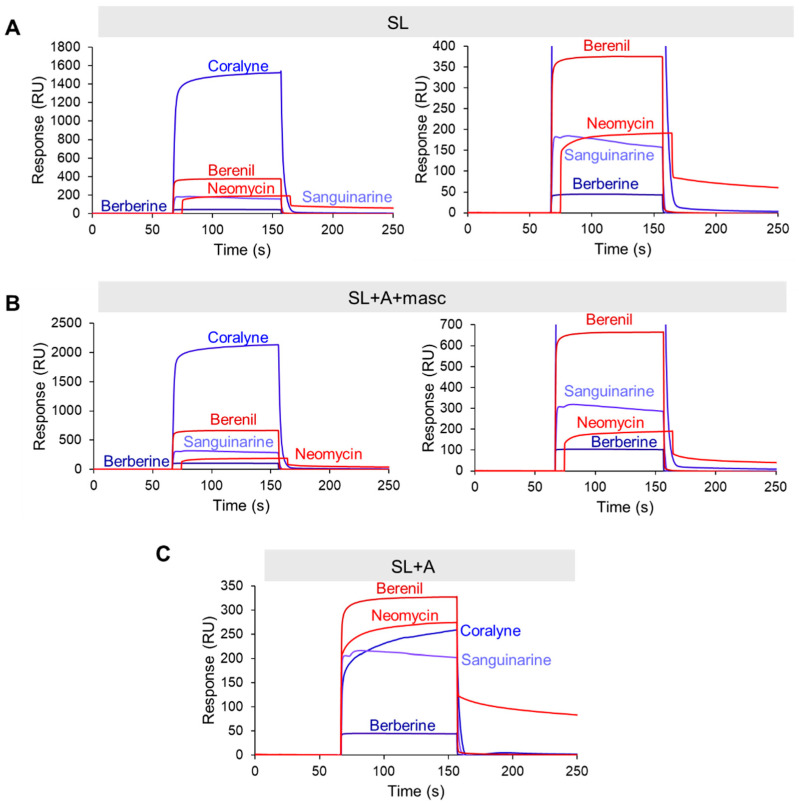
SPR sensogram plots for TBMs binding to the premature and mature MALAT1 RNAs. Representative SPR sensograms are shown for (**A**) SL, (**B**) SL+A+masc, and (**C**) SL+A. Association step lasted 90 s, corresponding to the 59.1 s to 151.1 s time points on the plots. The concentration of TBM was 100 µM. Right panels for A and B show sensograms for SL and SL+A+masc with max *y*-axis values at 400 and 700 RU, respectively. All SPR sensograms are presented in Figures S8 and S9. All raw sensogram data are available in File S3.

RNase P efficiently processes the 3′ end of MALAT1; therefore, any TBM targeting the precursor states would need to occur rapidly [10]. We employed surface plasmon resonance (SPR) to evaluate the relative binding trends of the TBMs engaging with the MALAT1 SL, SL+A+masc, and SL+A. We did not evaluate mascRNA, as the TBMs did not reveal a major impact on thermal stability (i.e., ∆*T*_M_ < 3.2 °C, Table S8). To perform this experiment, we first modified each RNA by extending the 5′ end with a 24-nucleotide sequence that is complementary to a 3′-biotinylated DNA oligonucleotide (Table S6). This biotinylated DNA-RNA hybrid was immobilized onto the streptavidin-coated SPR sensor chip, followed by the injection of TBMs (0, 1, 10, and 100 µM) (Figure S8). Binding trends were inferred at 100 µM TBM concentration to ensure steady-state and saturation of the RNA. Our UV melting results revealed that the flavonoids did not alter the thermal stability of MALAT1 SL+A (Figure 2C) and SL+A+masc (Figure 4H); therefore, we did not examine these TBMs. We analyzed the SPR sensograms to qualitatively examine the relative association and dissociation slopes for MALAT1 SL, SL+A+masc, and SL+A (Figure 5A–C and Figure S9). Changes in the slope of the association phase were used to rank the TBMs on binding speed. Quick binding interactions were defined as steep slopes lacking curvature, while slow binding was gradual slopes with rounded curvature. Based on these criteria, the relative rankings from fastest to slowest are the same for all three RNAs: berberine = sanguinarine > berenil > coralyne > neomycin (Figure 5A–C and Figure S9). Coralyne displays an unusually large response, which may indicate self-association [38]. Dissociation slopes were relatively steep and similar for all TBMs, although neomycin did not fully dissociate after 100 s (Figure S9E). These results show that the five TBMs interact transiently with the three different RNAs, suggesting that it is possible for the TBMs to dual target the premature and mature forms of the MALAT1 triple helix.

### 2.5. TBMs Reduce MALAT1 Levels More Than MENβ in HCT116 Cells

**Figure 6:**
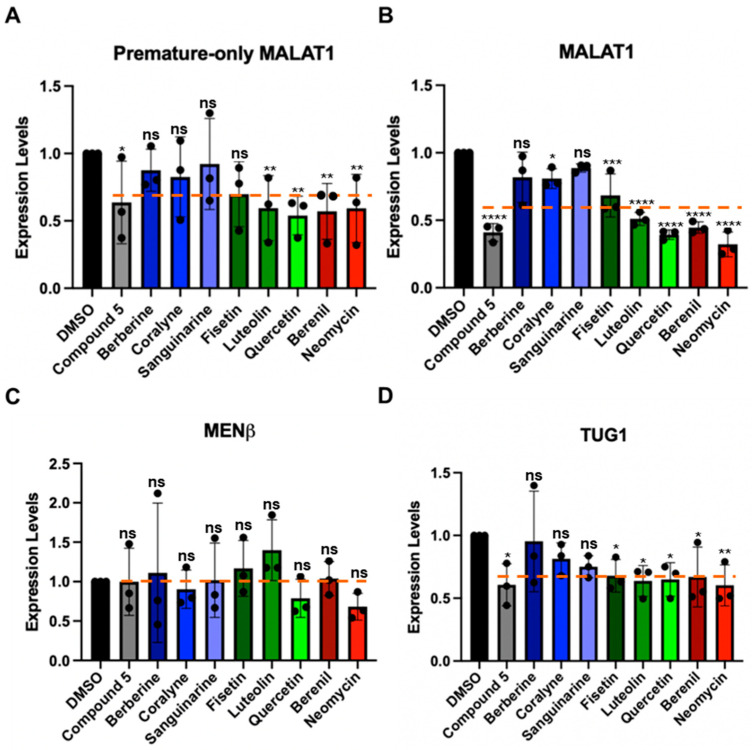
Effect of TBMs on the expression levels of various RNAs in HCT116 cells. RT-qPCR results show changes in expression for (**A**) premature-only MALAT1, (**B**) premature and mature MALAT1, (**C**) MENβ, and (**D**) TUG1 (unspliced Intron 1) when HCT116 cells were treated for 48 h with 1 μM of each TBM (berberine, coralyne, sanguinarine, fisetin, luteolin, quercetin, berenil, and neomycin) and with a previously studied TBM: compound **5** (Figure S5) [16]. The expression values were first normalized with respect to the geometric mean of four housekeepers: 18s rRNA, beta-actin (β-actin), glyceraldehyde-3-phosphate dehydrogenase (GAPDH), and U6 snRNA. The resulting values were then normalized with respect to the DMSO-treated sample set at an arbitrary value of 1 [58]. Results are averages ± standard deviation of biological triplicates (n = 3). *p*-values were calculated from the average 2^−∆∆CT^ using a two-way ANOVA test, comparing each TBM to the DMSO control: **** *p*-value < 0.0001, *** *p*-value < 0.0002, ** *p*-value < 0.0021, * *p*-value < 0.0332, ^ns^ *p*-value < 0.1234. The software used for statistical analysis was GraphPad Prism 10 (RRID:SCR_002798). An orange dashed line indicates average expression change for each RNA target across all TBMs. Raw and processed RT-qPCR data, including PCR efficiencies (90.1–98.7%), are presented in File S4.

Although the TBMs interact with MALAT1 SL+A and SL+A+masc in vitro (Figure 4 and Figure 5), we also determined if they could alter the levels of mature and premature MALAT1 inside cells. HCT116 cells were treated with 1 µM of each TBM because previously 1 µM of compound **5** (Figure S5) and 1 µM of quercetin (Figure 1D) were shown to decrease MALAT1 levels by ~50% in the mouse mammary tumor virus-polyoma middle tumor-antigen (MMTV-PyMT) tumor organoid model and MCF7 breast cancer cell lines, respectively [16,20]. Furthermore, an MTT assay showed no measurable cytotoxic effects when HCT116 cells were treated with 1 µM TBM (Figure S10 and File S4) [16]. First, we used a primer pair that will detect only premature MALAT1 (Figure 6A) and a primer pair that detects both the premature and mature forms of MALAT1 (Figure 6B, Table S9). The positive control, compound **5** (Figure S5), reduced MALAT1 levels by 50%, as observed previously in MMTV-PyMT tumor organoids, and had a similar effect on lowering premature-only MALAT1 (Figure 6A,B, Table S10) [16]. Except for the three alkaloids, the levels of mature and premature MALAT1 (Figure 6A,B, Table S10) were reduced by ~30–70% (Figure 6B, Table S10). The alkaloids had a more modest decrease in approximately ~20% (Figure 6B, Table S10). Additionally, quercetin reduced MALAT1 levels 60% in HCT116 cells (Figure 6B, Table S10), which is similar to the levels measured for MCF7 cells [20].

We next tested whether the TBMs altered the expression of MENβ, an lncRNA that ends in a putative triple helix akin to that of the MALAT1 SL+A, and the intron 1-retained TUG1 lncRNA, which currently does not have any known triple-helical stability elements; its expression levels are similar to the MALAT1 and MENβ lncRNAs, and unspliced TUG1 localizes in the nucleus (Table S11) [57]. None of the TBMs significantly decrease MENβ compared to MALAT1 (Figure 6B,C), and the TBMs exhibit a selectivity factor of 1.0 to 2.6 for mature MALAT1 over MENβ (Table S12). All TBMs, except for berberine, decreased the levels of unspliced TUG1 (Figure 6D, Table S10). In general, the average percent decrease in unspliced TUG1 (30%) was comparable to or slightly less than the percent decrease in mature and premature (40%) and premature-only (30%) MALAT1 (Table S10). Additionally, the percent decrease in all three RNAs was greater than for MENβ (0%) (Table S10), demonstrating slightly more selective targeting of MALAT1 by TBMs yet revealing off-target effects. Overall, these results demonstrate that flavonoids, berenil, and neomycin affect the expression of other lncRNA and premature-only MALAT1. However, there is still a larger effect on MALAT1, providing evidence that, amongst the TBMs tested herein, the flavonoids and berenil are the most promising scaffolds for selective targeting of the MALAT1 triple helix.

## 3. Discussion

MALAT1 has garnered tremendous research interest as a diagnostic biomarker and as a therapeutic target, particularly with the triple helix being a major drug target [5,14,15,16,17,18,19,20,21,22,34,59]. However, the molecular details of TBMs interacting with triple helices are comparatively less understood than small molecules binding to double-stranded DNA/RNA. Several factors influence how a small molecule recognizes RNA versus a protein, such as a high electronegative surface potential and a lack of structural diversity due to only four dominant nucleobases and a less pronounced binding pocket [60]. In general, small molecules that bind to RNA share the following features: heteroatom-rich aromatic rings, numerous hydrogen bond donors and acceptors, large polar surface area, low octanol-water partition coefficient, and 3D rod-like shape [60]. Thus far, the following small molecules have been investigated for their ability to interact with the MALAT1 triple helix: DPF derivatives [14,15], an imidazole-derived compound [16], a benzimidazole-derived compound [16], 1,2,3-triazole-derived compounds [18], an aromatic sulfonamide like MTC07 [19], berenil derivatives [17], and quercetin [20] (Figure 1D and Figure S5). Previous studies involving DPF and berenil derivatives demonstrated that small molecules with a 3D-rod shape, rather than sphere or disc shapes, prefer to bind to the MALAT1 triple helix and require 8–10 base triples for 1:1 binding [14,15,17,61]. In our study, we expanded this collection of small molecules with diverse classes of previously reported TBMs such as the alkaloids with polycyclic aromatic rings (berberine, coralyne, and sanguinarine); flavonoids rich in hydrogen bond donor and acceptor sites (fisetin, luteolin, and quercetin); triazene (berenil) with a 3D rod shape; and aminoglycoside (neomycin) with hydrogen bond donor and acceptor groups attached to aliphatic rings (Figure 1D) [24,25,26,28,29,30,31,32]. We determined that (i) berberine and berenil predominately stabilize only the WT MALAT1 SL+A (Figure 2 and Figure 4), (ii) alkaloids display greater sensitivity to the length of the MALAT1 triple helix, whereas base composition impacts the flavonoids (Figure 2D–H), (iii) most TBMs have a selectivity factor for MALAT1 over MENβ greater than 2, demonstrating selective targeting of the MALAT1 triple helix over the similar MENβ triple helix (Table S10), and (iv) neomycin shows mostly non-specific behavior with all MALAT1 RNAs (Figure 2, Figure 3, Figure 4, Figure 5 and Figure 6).

The alkaloid TBMs berberine and sanguinarine are naturally occurring, and coralyne is a synthetic analogue of berberine (Figure 1D). All three alkaloids preferentially stabilize the interactions that contribute to the Hoogsteen melting temperature of a poly(U•A-U) triple helix by ~3–17 °C and in the much shorter U•A-U triple helix of MALAT1 by ~2.5–7.8 °C (Figure 2C and Figure S11) [24,25,26]. Amongst all TBMs tested herein, the alkaloids are an attractive scaffold because of (i) sensitivity to length and nucleotide composition of the triple helix, suggesting fewer off-target effects (Figure 2), and (ii) their fast association and increased binding response for berberine and sanguinarine to the MALAT1 SL+A+masc and SL+A, suggesting the potential to dual-target the premature and mature forms of the MALAT1 triple helix (Figure 5). Their major disadvantage is their mild decrease in MALAT1 levels in HCT116 cells (Figure 6A,B), although their poor performance could be due to other factors.

All three flavonoids examined herein are naturally occurring and readily available as antioxidant dietary supplements (Table S5) [62,63,64]. The three flavonoid TBMs show selective stabilization for Hoogsteen melting (~14–17 °C) (Figure S11) over Watson-Crick (<5 °C) in the presence of poly(U•A-U) triple helices and/or poly(A-U) duplexes [28,29,30]. In contrast, all three flavonoids mildly destabilized Hoogsteen melting of the various MALAT1 triple helices, including the all U•A-U triple helix variant c, with Δ*T*_M,H_ and Δ*T*_M,WC_ values of approximately −3.7 °C (Figure 2C,F and Figure S11, Table S2). These different trends may be due to structural context because, unlike poly(U•A-U) triple helices, the major-groove triple helix of MALAT1 and variant c is flanked by various structures: double-stranded RNA, a GG bulge, and an A-minor motif (Figure 1B) [11,65]. The flavonoids are an interesting scaffold, as they demonstrate unique sensitivity to changes in nucleotide composition and A-minor disruptions compared to base triple length, which the flavonoids did not have a significant impact on the *T*_M,H_ for variant d-e (Figure 2D–J). FpocketR analysis shows unique binding pockets predominately in the duplex-triplex junction, where all three flavonoids are predicted to bind in our computational binding analysis (Figures S3 and S4). Quercetin was shown to bind along the major groove in silico, which is stabilized via electrostatic and hydrogen bond interactions with the MALAT1 triple helix [20]. Other studies reported intercalative binding interactions with poly(U•A-U), resulting in the stabilization of the RNA construct [29]. In this study, we observed destabilization for the poly(U•A-U) variant c as well as variants with increased C^+^•G-C base triples (Figure 2D–F). Unlike the poly(U•A-U) triple helices, MALAT1 is surrounded by duplexes and unstructured loops, which could explain the difference in binding trends. Alterations to the MALAT1 triple helix composition could modify base stacking and electrostatic interactions, possibly influencing the binding mode of the flavonoids and disruption of the RNA structure. Unlike the alkaloids in their iminium state [66], the flavonoids are not charged at neutral pH [29], which could influence the binding interactions when nucleotide composition is altered.

Berenil (diminazene aceturate) is one of the earliest known compounds tested for binding to poly (U•A-U or T•A-T) triple helices [31]. For the MALAT1 triple helix, berenil increases Hoogsteen melting more than Watson-Crick and does not alter the thermal stability of MALAT1 triple helix variants nor the premature forms of the MALAT1 triple helix (Figure 2 and Figure 4). Berenil has an established equilibrium dissociation constant of ~1.4 to 34.1 µM for poly(U•A-U) triple helices [17,31]. Differential scanning calorimetry (DSC) showed that binding of berenil is enthalpically driven for DNA/RNA triple helices but not the corresponding duplexes; a similar outcome was reported for berenil derivatives bound to the MALAT1 triple helix based on isothermal titration calorimetry results [17,31]. Our study, along with Zafferani et al., who used berenil-derived compounds to target the MALAT1 triple helix [17], shows that berenil alone, although more selective than most TBMs, is not sufficient to achieve selective binding to the MALAT1 triple helix but requires incorporating rod-like properties through chemical modifications.

Neomycin is an aminoglycoside antibiotic that binds in a pocket composed of noncanonical base pairs and nucleotide bulges of the A-site in *Escherichia coli* 16s rRNA [67]. Neomycin selectively increases Hoogsteen melting of 22-base triple U•A-U and T•A-T triple helices by approximately 4–25 °C (Figure S11), which is similar to our observed ∆*T*_M,H_ of ~31 °C for the MALAT1 triple helix (Figure 2 and Figure 4) [32]. Neomycin appears to have nonspecific interactions likely influenced by its positive charge (Figure 1D). Additionally, neomycin thermally stabilizes the Watson-Crick interface and has similar binding responses for premature and mature MALAT1 triple helixes (Figure 4H,I and Figure 5). Our study confirms the suspicion of others in the field: that aminoglycosides like neomycin are a poor choice to specifically target the MALAT1 triple helix [14,68].

Among all small molecules known to bind the MALAT1 triple helix (Figure 1D and Figure S5), the tightest binders are 1,2,3-triazole-derived compounds **3**, **7**, **10**, and **15** (*K*_D_ = 13 nM to 655 nM) [18] = diphenylfuran derivatives (EC_50_ = 27 nM to 1.6 µM) [14,15] > compound **5** (*K*_D_ = 2.3 µM) [16] > DMZ-M1 (*K*_D_ = 5.0 µM) [17] > compound **16** (*K*_D_ = 6.1 µM) [16] > quercetin (*K*_D_ = 0.5 µM) [20] > MTC07 (*K*_D_ = 400 µM) [19]. Although test-tube assays are informative, cell-based assays are needed to determine if small molecules can decrease MALAT1 levels, which is the desired outcome to treat most MALAT1-upregulated human diseases, particularly cancer. Six of the eight TBMs have been explored previously to treat cancer: berberine [69], coralyne [70], sanguinarine [71], fisetin [72], luteolin [62], quercetin [73], and berenil [74] (Table S5). Only neomycin reduced RNP formation in the competitive EMSAs (Figure 3), and most TBMs decreased premature and mature MALAT1 levels in treated HCT116 cells (Figure 6A,B). The maturation of the MALAT1 triple helix via RNase P cleavage is vital for the stabilization and the accumulation of MALAT1 [10]. This brings to question whether TBMs could recognize the MALAT1 SL+A+masc prior to RNase P cleavage, disrupting maturation and the accumulation of MALAT1. Our in vitro thermal denaturation and SPR assays suggest that most TBMs prefer binding to the MALAT1 triple helix and the precursor SL+A+masc rather than the U-rich SL (Figure 4 and Figure 5). Importantly, TBM binding is relatively rapid, suggesting berenil could decrease maturation efficiency via RNase P (Figure 5). However, cell-based assays showed berenil reducing mature and premature MALAT1 levels similarly (Figure 6A,B). Importantly, MENβ levels were not significantly reduced even though this lncRNA terminates in a triple helix that is predicted to be highly similar to that of MALAT1 (Figure 6C) [8,9]. Thus, it is not clear if TBMs reduce MALAT1 levels via direct interference with the triple helix or by other mechanisms. Additionally, we cannot rule out that the TBMs could be interacting with the peripheral structural motifs surrounding the MALAT1 triple helix.

To date, there are six TBMs whose selectivity factor for reducing MALAT1, but not MENβ, is at least two: luteolin (2.6), berenil (2.3), neomycin (2.1), compound **5** (~2.0–2.2) [16], compound **16** (~2.1) [16], and quercetin (~2.0) (Table S12) [20]. Comparing expression levels for only MALAT1 and MENβ provides an inadequate assessment of selectivity. Compound **5**, despite demonstrating selective targeting of MALAT1 over MENβ in MMTV-PyMT cells, also reduced unspliced TUG1 levels (Figure 6D, Table S10). No RNA-seq studies have been performed on human cells treated with any of the eight TBMs studied herein, but most TBMs appear to be promiscuous given their widespread pharmacological effects and disparate biological targets (Table S5). Additionally, TBM dose is important because compound **1a** for *DMPK* mRNA displays higher selectivity at higher doses [75]. In theory, developing a MALAT1-specific TBM should be achievable based on our results presented herein and published literature. One such example of a small molecule specifically targeting an RNA is Risdiplam, an *SMN2* splicing modulator compound, which causes minimal off-target effects on pre-mRNA splicing [76]. One important consideration is the cellular localization of small molecules inside cells. A recent study [77] showed that small molecules like berberine are enriched in the mitochondria, yet MALAT1 is predominantly in nuclear speckles [6] (Tables S5 and S11) [78,79,80,81,82,83,84,85,86,87,88,89,90,91,92,93,94,95,96,97,98,99,100,101,102,103,104,105,106,107,108,109,110,111,112,113,114,115,116,117,118,119,120,121,122,123,124,125,126,127,128]. Other studies show that sanguinarine [97], fisetin [129], and quercetin [130] are localized in the nucleus, but we did not observe any correlation between the percent decrease in nuclear lncRNAs like premature or mature MALAT1 [6], MENβ [131], and unspliced TUG1 [57] and TBM localization, likely because the net effect is a culmination of multiple factors such as cellular uptake of TBM, cellular localization of TBM, metabolism of TBM, free TBM concentration, RNA recognition, and off-target effects such as interactions with other cellular components that could impact RNA expression (Figure 6, Table S5). Our study demonstrates that TBMs reduced MALAT1 levels more than other nuclear lncRNAs, but further work is needed to improve TBM specificity, cellular localization, and selectivity of the MALAT1 lncRNA over other RNA types.

In summary, this study expands our characterization-knowledge base of TBMs that interact with the MALAT1 triple helix. In addition to compound **5**, DPFp8, berenil, and its derivatives (Figure 1D and Figure S5), TBMs like alkaloids (with extended aromatic π systems) and flavonoids (with rich hydrogen bond donor and acceptor sites) may represent a suitable scaffold but would benefit greatly from a 3D structure of the MALAT1 triple helix in complex with a TBM. Other classes that remain underexplored include ruthenium and other metal complexes [132], polyamines [133], electrophilic covalent inhibitors [134], and TBMs conjugated to oligonucleotides [135]. Another intriguing possibility would be synthetic cyclic mismatch-binding ligands that bind to RNA via pseudo-canonical base pairs [136]. Additionally, human health and medicine, small molecules that bind to double-stranded RNA, such as psoralen [137], and G-quadruplexes, such as QUMA-1 [138], represent research tools that have expanded our knowledge of the biological and cellular roles of non-canonical structures of nucleic acids. A TBM with exquisite specificity for triple helices could do the same.

## 4. Materials and Methods

### 4.1. Preparation of RNA

DNA oligonucleotides were chemically synthesized by Sigma-Aldrich (St. Louis, MO, USA); these oligonucleotides were used in PCR reactions to generate the DNA template needed for in vitro transcription reactions. Wild-type MALAT1 triple helix, extended MALAT1 triple helix, and MALAT1 triple helix variants (Table S6) were generated via in vitro transcription using homemade T7 RNA polymerase as previously described [9]. RNAs were gel purified, treated with phenol-chloroform (Thermo Fisher Scientific, Waltham, MA, USA), and precipitated using isopropanol (JT Baker, Radnor, PA, USA). The final RNA concentrations were measured at room temperature using a NanoDrop^TM^One^C^ (Thermo Fisher Scientific, Waltham, MA, USA) to determine absorbance at 260 nm. For the competitive EMSAs, the 5′ end of the MALAT1 triple helix was dephosphorylated using calf intestinal alkaline phosphatase (CIP) (New England Biolabs, Ipswich, MA, USA) and radiolabeled using γ-[^32^P]ATP (~7000 Ci/mmol, PerkinElmer, Shelton, CT, USA) and T4 PNK (New England Biolabs, Ipswich, MA, USA) per the manufacturer’s protocol. G25 microspin columns (GE Healthcare, Chicago, IL, USA) were used to remove excess γ-[^32^P]ATP.

### 4.2. Triplex-Binding Molecules

All triplex-binding molecules (TBMs) were purchased from various commercial vendors with >90% purity and used as such. Berberine, berenil, compound **5** (i.e., MALAT1-IN-5), coralyne, neomycin, quercetin, and sanguinarine were purchased from Sigma-Aldrich (St. Louis, MO, USA); fisetin was purchased from PhytoLab (GmbH & Co., Vestenbergsgreuth, Germany); and luteolin was acquired from Indofine chemicals (Hillsborough, NJ, USA). Water-soluble compounds (i.e., berberine, berenil, coralyne, and neomycin) were dissolved in deionized autoclaved water. Stock solutions for fisetin, luteolin, quercetin, sanguinarine, and compound **5** were made by dissolving molecules in 100% DMSO (AmericanBio, Canton, MA, USA) and further diluted with deionized water until the final DMSO concentration was 0.1%. Unless stated otherwise, 0.1% DMSO was maintained in all the samples irrespective of solubility.

### 4.3. UV Thermal Denaturation Assay

A Cary 3500 Multicell UV-Vis Spectrophotometer (Agilent Technologies, Santa Clara, CA, USA) was used to conduct all the UV thermal denaturation assays along with quartz cuvettes (Starna Cells, Inc., Atascadero, CA, USA) having an optical path length of 1 cm. For each sample, the total RNA concentration (with or without TBMs) was maintained at 0.5 μM. TBM concentration was 10 µM (i.e., RNA:TBM stoichiometry was 1:20). All RNA absorbances (0.3–2.1, see File S1) were measured within the linear range (<6) of the Cary 3500 Multicell UV-Vis Spectrophotometer [139]. RNA samples were prepared in low ionic UV buffer (25 mM sodium cacodylate pH 7.0 (Sigma-Aldrich, St. Louis, MO, USA), 50 mM KCl (Sigma-Aldrich, St. Louis, MO, USA), 0.1 mM MgCl_2_ (Sigma-Aldrich, St. Louis, MO, USA), and 0.1% DMSO) and were folded by heating (25 °C to 95 °C) and cooling (95 °C to 25 °C) at a ramp rate of 5 °C/min. TBMs were added (maintaining the final DMSO concentration at 0.1% except when 2% DMSO was used for flavonoids) soon after the folding step and incubated for an additional 30 min at 25 °C. Absorbance was measured at 260 nm, which is the same wavelength used for previous MALAT1 triple helix thermal denaturation assays [9,14,20,33,59,65,140]. Absorbance was recorded at 0.3 °C intervals from 25 °C to 95 °C at a ramp rate of 0.8 °C/min. All the melting curves were buffer subtracted, and the melting temperatures were extrapolated from the peak maxima of first derivatives of the melting curves (δA/δT) that were smoothed over 1.2 °C using the Savitzky-Golay method. Raw and processed data are provided in File S1.

### 4.4. Predicting RNA-Ligand Complexes and FpocketR

RNA-ligand complex predictions for TBMs bound to the MALAT1 triple helix crystal structure (PDB ID: 4plx) (nts 1-76) were generated via AlphaFold3 (AlphaFold3 inference pipeline v3.0.1) [40]. Inputs were the 4plx RNA FASTA and TBM smiles, which are included in File S2. The PDB complex output files were analyzed using PyMOL (v. 4.6.0, PyMOL. Retrieved from http://www.pymol.org/pymol; accessed on 23 September 2025).

FpocketR software (version 1.3.4) was used to compute potential binding pockets of the MALAT1 variants d-e (sequences are provided in Table S7) [43,44]. The MALAT1 triple helix variants d-e were generated using AlphaFold 3 via the AlphaFold 3 (AlphaFold3 inference pipeline v3.0.1) and exported as PDBs. The input sequences are those shown in Figure 2G,H. These RNAs were then used as the input for FpocketR software (version 1.3.4). Visual presentation of these binding pockets was completed using PyMOL (v. 4.6.0).

### 4.5. SPR

This SPR protocol was adopted from previously published methods [141,142,143]. All SPR experiments were conducted using a Biacore^TM^ T200 (Cytiva Life Sciences, Marlborough, MA, USA) surface plasmon resonance system with a high-affinity streptavidin (SA) sensor chip (Cytiva Life Sciences, Marlborough, MA, USA) to immobilize biotinylated molecules for SPR interaction analysis. The MALAT1 SL, MALAT1 SL+A+masc, and MALAT1 SL+A have a 5′-24-nucleotide extension (Table S6) that was annealed to a complementary 3′-biotinylated DNA oligonucleotide (5′-TTCACAGTGGCTAAGTTCCGC-3′ (Sigma-Aldrich, St. Louis, MO, USA)). RNA was folded using a high-ionic SPR buffer (10 mM Tris pH 7.5 (Thermo Fisher Scientific, Waltham, MA, USA), 1 mM MgCl_2_, 152.6 mM NaCl (Sigma-Aldrich, St. Louis, MO, USA)). Before immobilization, the SA sensor chips were activated using 50 mM NaOH and 1 M NaCl, followed by priming the sensor chip with the high-ionic SPR running buffer. For the immobilization, 25 nM of the extended RNA and 25 nM of the 3′-biotinylated oligonucleotide (1:1 stoichiometry) were folded by heating to 95 °C for 5 min, snap cooling on ice for 10 min, followed by incubation at room temperature for 1.5 h. After attaining the required baseline stabilization, the annealed biotinylated DNA-RNA hybrid was immobilized onto the streptavidin chip with a flow rate of 1 μL/min for about 100 min. The SPR running buffer was the high-ionic SPR buffer supplemented with 0.005% Tween-20 (AmericanBio, Inc., Canton, MA, USA) and 3% DMSO. After the immobilization step, varying concentrations of TBMs (0, 1, 10, and 100 μM, from low to high concentration to avoid carryover artifacts from high TBM concentrations) (Figure S8) were injected with a 90-s association step and varying dissociation times (350 to 1000 s) dependent on test injections at a flow rate of 50 μL/min. A reference subtraction method was used for each TBM injection, which corresponds to the subtraction from the empty reference flow cell. Raw and processed data are in File S3.

### 4.6. Culturing HCT116 Cells

Human colorectal carcinoma (HCT116) cells (RRID:CVCL_0291) were grown in complete McCoy’s 5A (modified) media (Thermo Fisher Scientific, Waltham, MA, USA) supplemented with 10% fetal bovine serum (Sigma-Aldrich, St. Louis, MO, USA), 2 mM glutamate, and 1× penicillin-streptomycin (Thermo Fisher-Scientific, Waltham, MA, USA) at 37 °C with 5% CO_2_.

### 4.7. Preparation of Native HCT116 Whole-Cell Lysate

HCT116 cells (3 × 100-mm tissue culture plates) were harvested at ~90% confluency. After trypsinization, cells were centrifuged at 1200 rpm for 5 min. Cell pellets were resuspended in 500 µL native lysate buffer (50 mM Tris pH 7 at room temperature, 100 mM KCl, 0.2 mM EDTA (Sigma-Aldrich, St. Louis, MO, USA), 1 mM MgCl_2_, 10% glycerol (AmericanBio., Canton, MA, USA), 1 mM DTT, 1× EDTA-free protease inhibitor cocktail (Sigma-Aldrich, Roche Diagnostics GmbH, Mannheim, Germany), and 1 mM PMSF (AmericanBio, Canton, MA, USA)). Cells were sonicated 3 times for 7 s with 30-s intervals on ice. Lysed cells were then centrifuged at 4 °C at maximum RPM for 10 min. A BCA assay (ThermoFisher Scientific, Waltham, MA, USA) was used to determine the total protein concentration.

### 4.8. Competitive EMSA

All the samples were prepared in 1× EMSA buffer (25 mM HEPES pH 7.5 at room temperature (Sigma-Aldrich, St. Louis, MO, USA), 50 mM NaCl, 100 mM KCl, 1 mM MgCl_2_, 1 mM TCEP (Gold Biotechnology, St. Louis, MO, USA), 7% glycerol, 0.1% DMSO, 1 mg/mL yeast tRNA, and 2 nM MALAT1 SL+A). The 5′-[^32^P] radiolabeled MALAT1 SL+A was folded by heating at 95 °C (5 min) and snap cooling on ice (10 min), followed by equilibration at room temperature for 1 h. To the folded RNA, increasing amounts of TBMs (0–200 μM) were added and incubated at room temperature for 30 min. Then, cell lysate was added (∼1 μg/μL unless indicated otherwise) and incubated for an additional 30 min at room temperature. Using a 5% native polyacrylamide gel (19:1 acrylamide:bisacrylamide, 40 mM Tris-borate pH 8.3, 1 mM MgCl_2_), the samples were loaded and electrophoresed with running buffer (40 mM Tris-borate pH 8.3, 1 mM MgCl_2_) at 130 V for ∼3 h at room temperature. After wrapping the gel in a transparent plastic wrap, it was exposed to a Phosphorimager screen overnight. The screens were scanned using an Amersham Typhoon IP Phosphorimager 1.0.0. (GE Healthcare, Chicago, IL, USA).

### 4.9. Treatment of HCT116 Cells with TBMs and RT-qPCR

HCT116 cells were plated at a seeding density of 1.0 × 10^5^ cells/well in a 24-well plate and were grown to ~70% confluency. Each biological replicate was plated from different starting cells, and the same treatment was conducted for each replicate. After 48 h, cells were treated with 0.1% DMSO (AmericanBio, Canton, MA, USA) as a control or 1 µM of each TBM. Final DMSO was 0.1% for all compounds. After 48 h of treatment, cells were washed using 1× PBS (Sigma-Aldrich, St. Louis, MO, USA) and detached by applying 1 mL TRIzol (Life Technologies, Carlsbad, CA, USA) to each well. RNA was then extracted per the manufacturer’s recommended protocol. RNA was resuspended in 20 µL RNase-free water. RNA concentration was obtained at a 1:10 dilution using a NanoDrop^TM^ One/One^C^ Microvolume UV-Vis Spectrophotometer (Thermo Fisher Scientific, Waltham, MA, USA). RNA quality control was examining rRNA bands via a 1% agarose gel. DNase treatment and cDNA synthesis were performed using the iScript^TM^ gDNA Clear cDNA Synthesis Kit (Bio-Rad, Hercules, CA, USA) per the manufacturer’s instructions. Previously published primer pairs were used for premature and mature MALAT1 [12], MENβ [12], TUG1 [57], 18s rRNA [144], β-actin [145], U6 snRNA [146], and glyceraldyhde-3-phosphare dehydrogenase (GAPDH) [147] (Table S9) (Sigma-Aldrich, St. Louis, MO, USA). No template control, no reverse transcriptase control, and no standard curves were included. The amount of cDNA dilution used for all samples was determined based on Ct values for GAPDH at serial 10-fold dilutions. SsoAdvanced Universal SYBR^®^ Green Supermix (Bio-Rad, Hercules, CA, USA), 500 nM primer pairs (Sigma-Aldrich, St. Louis, MO, USA), and cDNA template (20 µL total volume) were combined in a 96-well plate, inserted into a Bio-Rad CFX96 Touch Real-Time PCR or Bio-Rad CFX Opus 96 (Bio-Rad, Hercules, CA, USA), and subjected to the following cycling conditions: activation: 98 °C for 3 min; denaturation/annealing: 98 °C for 5 s, 59.4 °C for 30 s (35 cycles); extension: 72 °C for 2 min). The 2^−ΔΔCT^ values [148] for premature-only MALAT1, premature and mature MALAT1, MENβ, and unspliced TUG1 RNAs were calculated using individual ΔCT values after normalization to the geometric mean of the four housekeepers, 18s rRNA, β-Actin, GAPDH, and U6 snRNA using GraphPad Prism 10. (RRID:SCR_002798) [58]. *p*-values were calculated from the average 2^−∆∆CT^ using a two-way ANOVA test, comparing each TBM to the DMSO control: **** *p*-value < 0.0001, *** *p*-value < 0.0002, ** *p*-value < 0.0021, * *p*-value < 0.0332, ^ns^ *p*-value < 0.1234. Raw and processed data, including PCR efficiencies (90.1–98.7%), for RT-qPCR experiments are in File S4.

### 4.10. MTT Assay

HCT116 cells were plated at a seeding density of 5000 cells/well in a 96-well plate. Following 48 h, cells were treated with 1 µM TBM in technical triplicates. After a 48-h treatment, media was removed, and fresh media (100 µL) and 5 mg/mL stock of 3-(4,5-dimethylthiazol-2-yl)-2,5-diphenyltetrazolium bromide (MTT) (10 µL) were added to each well (total volume 110 µL). After a 4-h incubation with MTT, 10% sodium dodecyl sulfate (SDS) (90 µL) and 100% DMSO (10 µL) were combined and added to each well (total volume 100 µL) and incubated at 37 °C overnight. After incubation, absorbance of each well is measured at a 590 nm wavelength using a Synergy H1 Microplate Reader (Agilent BioTek). Measurements are background subtracted using wells that contain only a mixture of 0.2 mg/mL MTT (0.2 mg/mL), 4.3% SDS, and 4.8% DMSO in media. After background subtraction, sample measurements are normalized to the control wells (cells + media). Data is an average of biological replicates (n = 3). Raw and processed data for the MTT assay are in File S4.

## Figures and Tables

**Table 1:** UV melting temperatures obtained for the MALAT1 triple helix in the absence or presence of TBMs.

TBM	*T*_M,H_ (°C)	Δ*T*_M,H_ (°C)	*T*_M,WC_ (°C)	Δ*T*_M,WC_ (°C)
0.1% DMSO	49.6 ± 0.4	-	69.0 ± 0.0	-
Berberine	53.3 ± 0.8	3.7	70.8 ± 0.4	1.8
Coralyne	52.1 ± 0.5	2.5	69.8 ± 0.3	0.8
Sanguinarine	57.4 ± 0.7	7.8	72.2 ± 0.4	3.2
Fisetin	49.1 ± 0.1	−0.5	69.0 ± 0.2	0.0
Luteolin	49.4 ± 0.5	−0.2	68.8 ± 0.4	−0.2
Quercetin	49.0 ± 0.1	−0.6	68.9 ± 0.3	−0.1
Berenil	54.1 ± 0.9	4.5	70.8 ± 0.4	1.8
Neomycin	80.3 ± 0.6	30.7	89.2 ± 0.4	20.2

The *T*_M_ values are the average ± standard deviation of three independent melting experiments.

## Data Availability

The original contributions presented in this study are included in the article/Supplementary Material. Further inquiries can be directed to the corresponding author.

## Supplementary Materials

The following supporting information can be downloaded at: https://www.mdpi.com/article/10.3390/molecules30214277/s1, Figure S1: UV thermal melting results for the TBMs. Figure S2: UV thermal assays performed using 2% DMSO for flavonoids. Figure S3: Prediction of MALAT1 triple helix-TBM complex. Figure S4: FpocketR analysis of the MALAT1 triple helix variants d and e. Figure S5: Chemical structures of previously reported TBMs binding to the MALAT1 triple helix. Figure S6: UV thermal melting results for the pre-mascRNA. Figure S7: UV thermal melting results for the MALAT1 RNA in the absence and presence of TBMs. Figure S8: SPR sensogram plots comparing how each TBM binds with the three different RNAs at all concentrations. Figure S9: SPR sensogram plots comparing how each TBM interacts with the three different RNAs. Figure S10: MTT cell viability assay for TBM-treated HCT116 cells. Figure S11: Bar plot showing Δ*T*_M,H_ values for the MALAT1 triple helix (solid color) and the poly(U•A-U) triple helix (light gray) in the presence of TBMs. Table S1: *T*_M_ values for the MALAT1 triple helix variants a–c in the absence or presence of TBMs. Table S2: Δ*T*_M_ values for the MALAT1 triple helix variants a–c in the absence or presence of TBMs. Table S3: *T*_M_ values for the MALAT1 triple helix variants d–g in the absence or presence of TBMs. Table S4: Δ*T*_M_ values for the MALAT1 triple helix variants d–g in the absence or presence of TBMs. Table S5: Summary of characteristics for each TBM and its subcellular localization. Table S6: In vitro transcribed RNAs used in this study. Table S7: Average *T*_M_ values for the premature and mature MALAT1 RNAs in the absence or presence of TBMs. Table S8: Δ*T*_M_ values for the premature and mature MALAT1 RNAs in the absence or presence of TBMs. Table S9: Sequences of primers used for RT-qPCR experiments. Table S10: Average 2^−∆∆CT^ values and standard deviation for all lncRNA targets. Table S11: Summary of lncRNA expression levels and cellular localization. Table S12: Selectivity factor of each TBM for reducing MALAT1 over MENβ. File S1: Raw and processed data from UV thermal denaturation experiments. File S2: FASTA and SMILES inputs for AlphaFold 3 complex prediction. File S3: Raw and processed data from SPR experiments. File S4: Raw and processed data from MTT and RT-qPCR experiments.

## Abbreviations

The following abbreviations are used in this manuscript:

DPF	diphenyl furan
DSC	differential scanning calorimetry
EMSA	electrophoretic mobility shift assay
GAPDH	glyceraldehyde-3-phosphate dehydrogenase
HCT116	human colorectal carcinoma cell line
lncRNA	long non-coding RNA
mascRNA	MALAT1-associated small cytoplasmic RNA
MALAT1	metastasis-associated lung adenocarcinoma transcript 1
MALAT1 SL	MALAT1 U-rich stem loop
MALAT1 SL+A+masc	MALAT1 U-rich stem loop, A-rich tract, and mascRNA
MALAT1 SL+A	MALAT1 U-rich stem loop and A-rich tract
MENβ	multiple endocrine neoplasia-β
METTL16	methyltransferase-like protein 16
MMTV-PyMT	mouse mammary tumor virus-polyoma middle tumor-antigen
MTT	3-(4,5-dimethylthiazol-2-yl)-2,5-diphenyltetrazolium bromide
RNP	ribonucleoprotein
RT-qPCR	quantitative reverse transcription polymerase chain reaction
SDS	sodium dodecyl sulfate
SPR	surface plasmon resonance
TBM	triplex-binding molecule
TUG1	taurine-upregulated gene
*T*_M_	melting temperature
∆*T*_M_	change in melting temperature or thermal shift
WT	wild type
