# Supplementary material for: Eight Triplex-Binding Molecules from Four Chemical Classes Broadly Recognize the MALAT1 Triple Helix
Source: Molecules. 2025 Nov 3;30(21):4277. doi: 10.3390/molecules30214277 (PMC12609000; doi:10.3390/molecules30214277)
Supplement: Supplementary file 1 [file molecules-30-04277-s001.zip › molecules-3934539-supplementary.pdf]

SUPPLEMENTARY MATERIALS

Eight Triplex-Binding Molecules from Four Chemical Classes Broadly Recognize the MALAT1 Triple Helix

Madeline M. Mousseau, Krishna M. Shivakumar, Jaesang Yoo, and Jessica A. Brown

**Figure S1**	UV thermal melting results for TBMs.
**Figure S2**	UV thermal assays performed using 2% DMSO for flavonoids.
**Figure S3**	Prediction of MALAT1 triple helix-TBM complex.
**Figure S4**	FpocketR analysis of the MALAT1 triple helix variants d and e.
**Figure S5**	Chemical structures of previously reported TBMs binding to the MALAT1 triple helix.
**Figure S6**	UV thermal melting results for the pre-mascRNA.
**Figure S7**	UV thermal melting results for the MALAT1 RNA in the absence and presence of TBMs.
**Figure S8**	SPR sensogram plots comparing how each TBMs bind with the three different RNAs at all concentrations.
**Figure S9**	SPR sensogram plots comparing how each TBM interact with the three different RNAs.
**Figure S10**	MTT cell viability assay for TBM-treated HCT116 cells.
**Figure S11**	Bar plot showing $\Delta T_{M,H}$ values for the MALAT1 triple helix (solid color) and the poly(U•A-U) triple helix (light gray) in the presence of TBMs.
**Table S1**	T_M values for the MALAT1 triple helix variants a-c in the absence or presence of TBMs.
**Table S2**	ΔT_M values for the MALAT1 triple helix variants a-c in the absence or presence of TBMs.
**Table S3**	T_M values for the MALAT1 triple helix variants d-g in the absence or presence of TBMs.
**Table S4**	ΔT_M values for the MALAT1 triple helix variants d-g in the absence or presence of TBMs.
**Table S5**	Summary of characteristics for each TBM and its subcellular localization.
**Table S6**	*In vitro* transcribed RNAs used in this study.
**Table S7**	Average T_M values for the premature and mature MALAT1 RNAs in the absence or presence of TBMs.
**Table S8**	ΔT_M values for the premature and mature MALAT1 RNAs in the absence or presence of TBMs.
**Table S9**	Sequences of primers used for RT-qPCR experiments.
**Table S10**	Average $2^{-\Delta\Delta CT}$ values and standard deviation for all lncRNA targets.
**Table S11**	Summary of lncRNA expression levels and cellular localization.
**Table S12**	Selectivity factor of each TBM for reducing MALAT1 over MENβ.
**File S1**	Raw and processed data from UV thermal denaturation experiments.
**File S2**	FASTA and SMILES inputs for AlphaFold 3-predicted RNA-TBM complexes.
**File S3**	Raw and processed data from SPR experiments.
**File S4**	Raw and processed data from MTT and RT-qPCR experiments.

SUPPLEMENTARY FIGURES

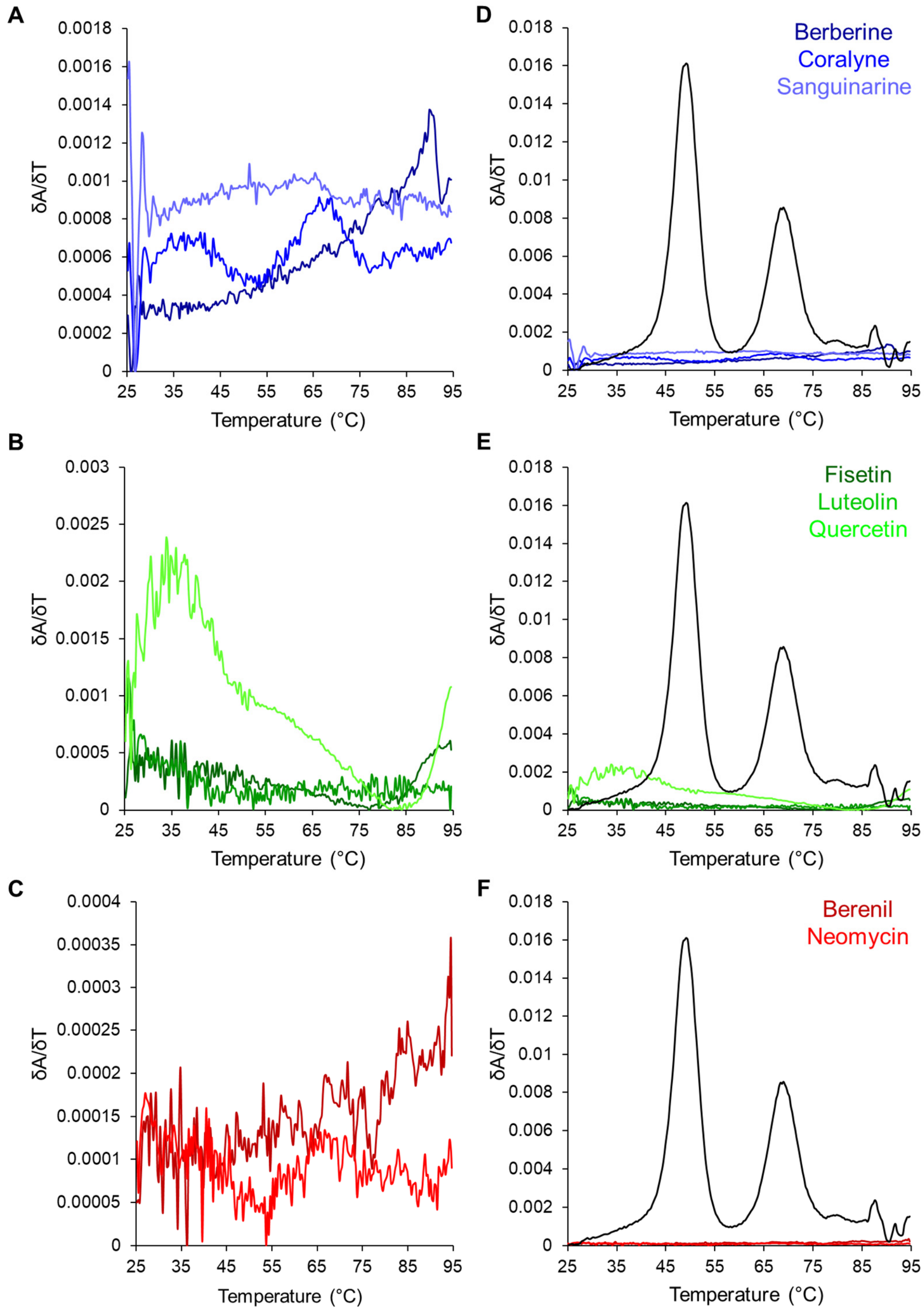

Figure S1. UV thermal melting results for TBMs. Plots of first derivatives of absorbance with respect to temperature versus temperature are shown for (A-C) only the TBMs and (D-F) the TBM-only results alongside the MALAT1 triple helix. This figure corresponds to **Figure 2**. Raw and processed data are presented in **File S1**.

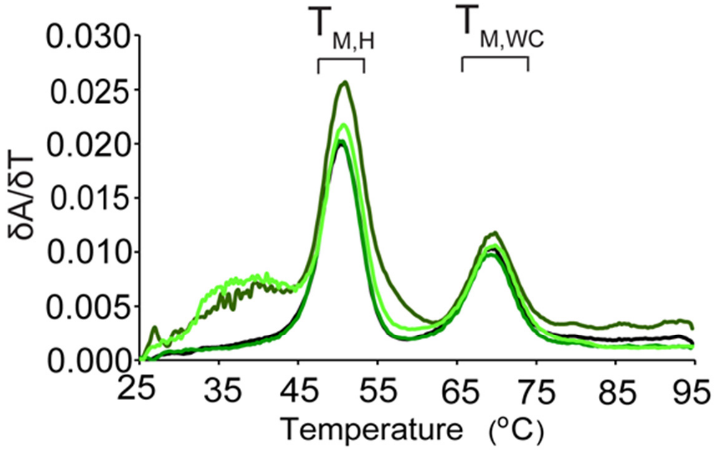

TBMs	$T_{M,H}$	$\Delta T_{M,H}$	$T_{M,WC}$	$\Delta T_{M,WC}$
2% DMSO	50.5 ± 0.1	-	69.5 ± 0.1	-
Fisetin	50.6 ± 0.4	0.1	69.7 ± 0.3	0.2
Luteolin	50.6 ± 0.4	0.1	69.5 ± 0.1	0.0
Quercetin	50.5 ± 0.1	0.1	69.9 ± 0.1	0.4

Figure S2. UV thermal assays performed using 2% DMSO for flavonoids. The UV thermal denaturation results are shown for the MALAT1 triple helix in the absence (black line) or presence of flavonoids (green shades) at 2% DMSO. Profiles are shown for first derivatives with respect to temperature versus temperature for the following TBMs: fisetin (dark green), luteolin (green) and quercetin (light green). The brackets denote two distinct peaks: the Hoogsteen ($T_{M,H}$) and the Watson-Crick ($T_{M,WC}$) melting temperatures. The values are the average ± standard deviation of three independent melting experiments. This figure corresponds to **Figure 2**. Raw and processed data are presented in **File S1**.

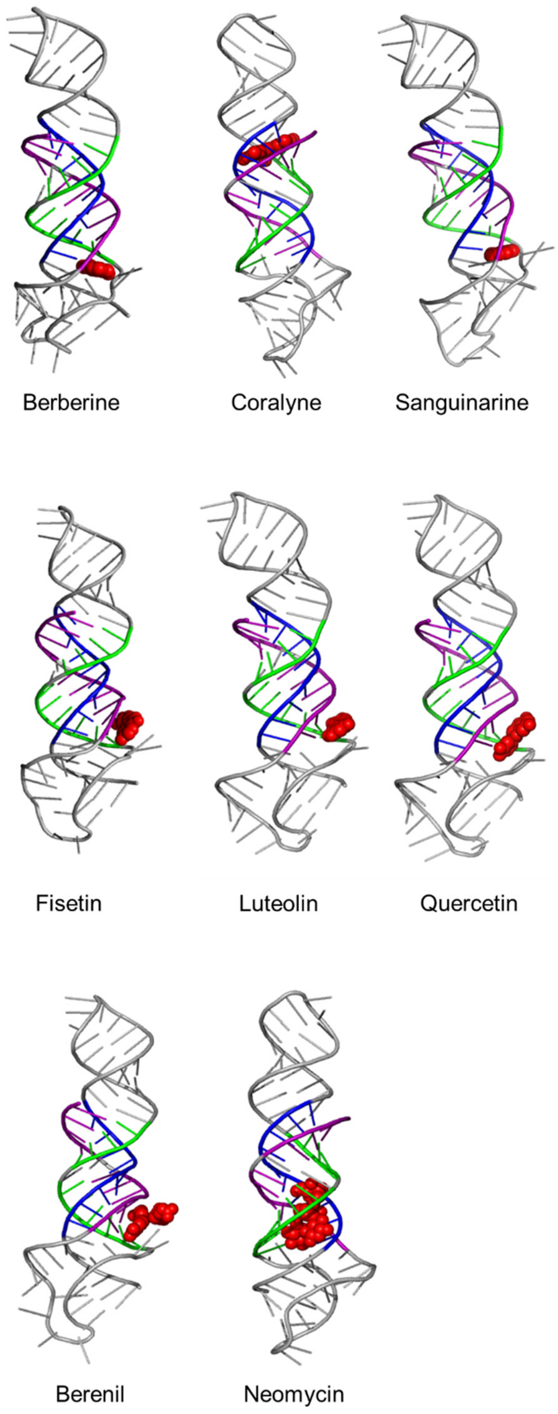

Figure S3. Prediction of MALAT1 triple helix-TBM complex. AlphaFold 3 [1] was used to predict the binding site of each TBM (red surface representation) with the 3D structure of the MALAT1 triple helix crystal structure (PDB ID: 4plx) [2], which is shown in cartoon mode. Colors represent nucleotides in the following strands of the major-groove triple helix: Hoogsteen (blue), Watson (purple), and Crick (green). Orientation of all 3D structures matches orientation shown in Figure 1B: stem II and I are respectively above and below the major-groove triple helix. FASTA and SMILES inputs are available in **File S2**.

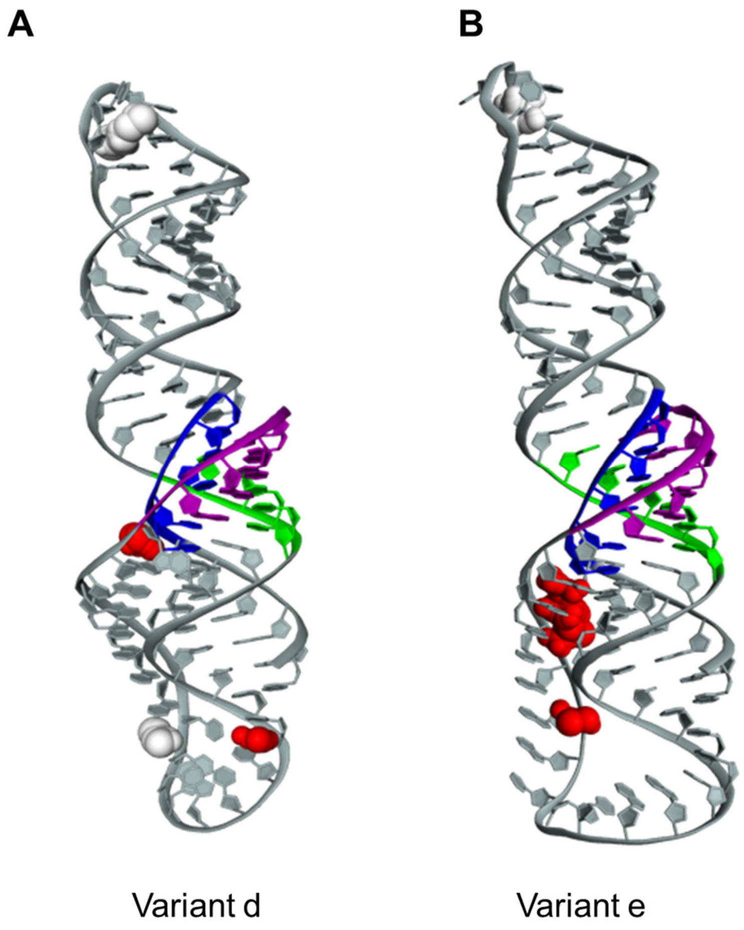

Figure S4. FpocketR [3] analysis of the MALAT1 triple helix variants d and e. The 3D structure of the MALAT1 triple helix variants d and e were generated using AlphaFold 3 [1]. FpocketR outputs are shown for (A) variant d and (B) variant e. Colors represent the following: Hoogsteen strand (blue), Watson strand (purple), Crick strand (green), similar binding pockets in both variants d and e (white) and unique binding pockets (red).

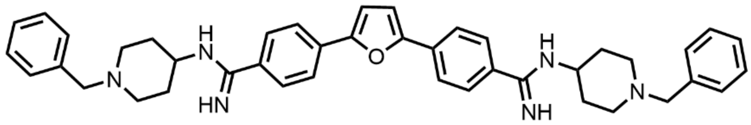

DPFp8

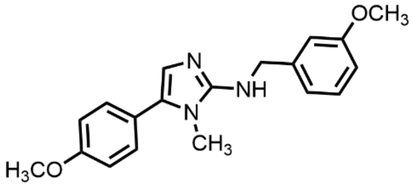

Compound 5

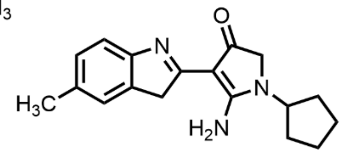

Compound 16

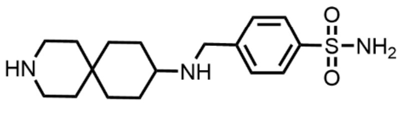

MTC07

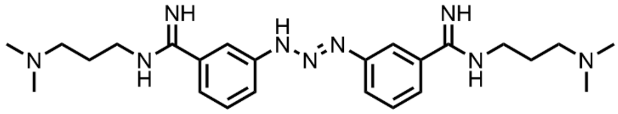

DMZ-M1

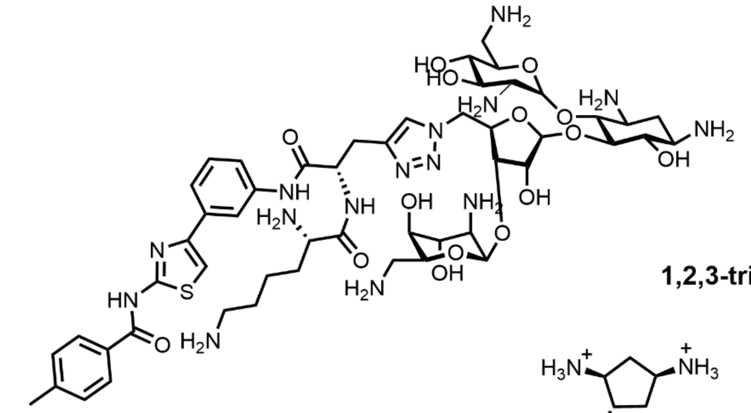

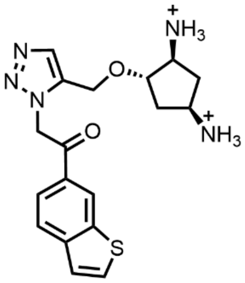

1,2,3-triazole derived compound 7

1,2,3-triazole derived compound 3

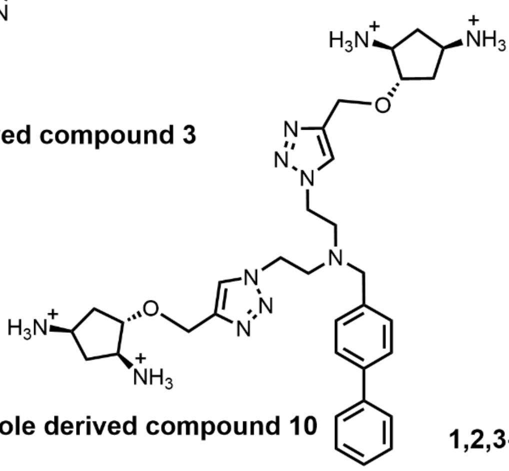

1,2,3-triazole derived compound 10

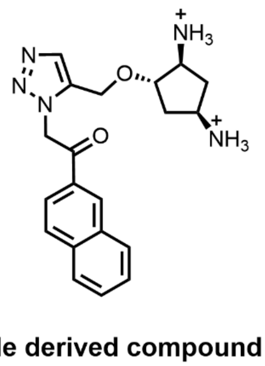

1,2,3-triazole derived compound 15

Figure S5. Chemical structures of previously reported TBMs binding to the MALAT1 triple helix [4–9]. Order corresponds to the order in which the TBMs binding to the MALAT1 triple helix were published.

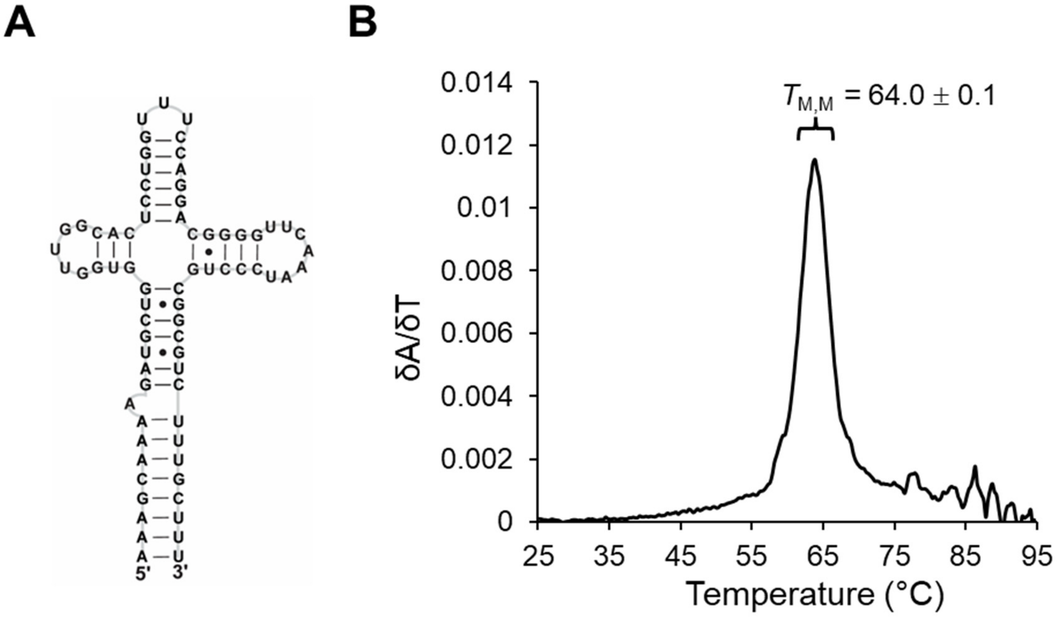

Figure S6. UV thermal melting results for the pre-mascRNA. (A) A schematic depicts the secondary structure of pre-mascRNA. (B) A plot of first derivative of absorbance with respect to temperature versus temperature for the pre-mascRNA in the absence of a TBM. The melting temperature ($T_{M,M}$) is the average ± standard deviation of three independent melting experiments. This figure corresponds to **Figure 4**. Raw and processed data are presented in **File S1**.

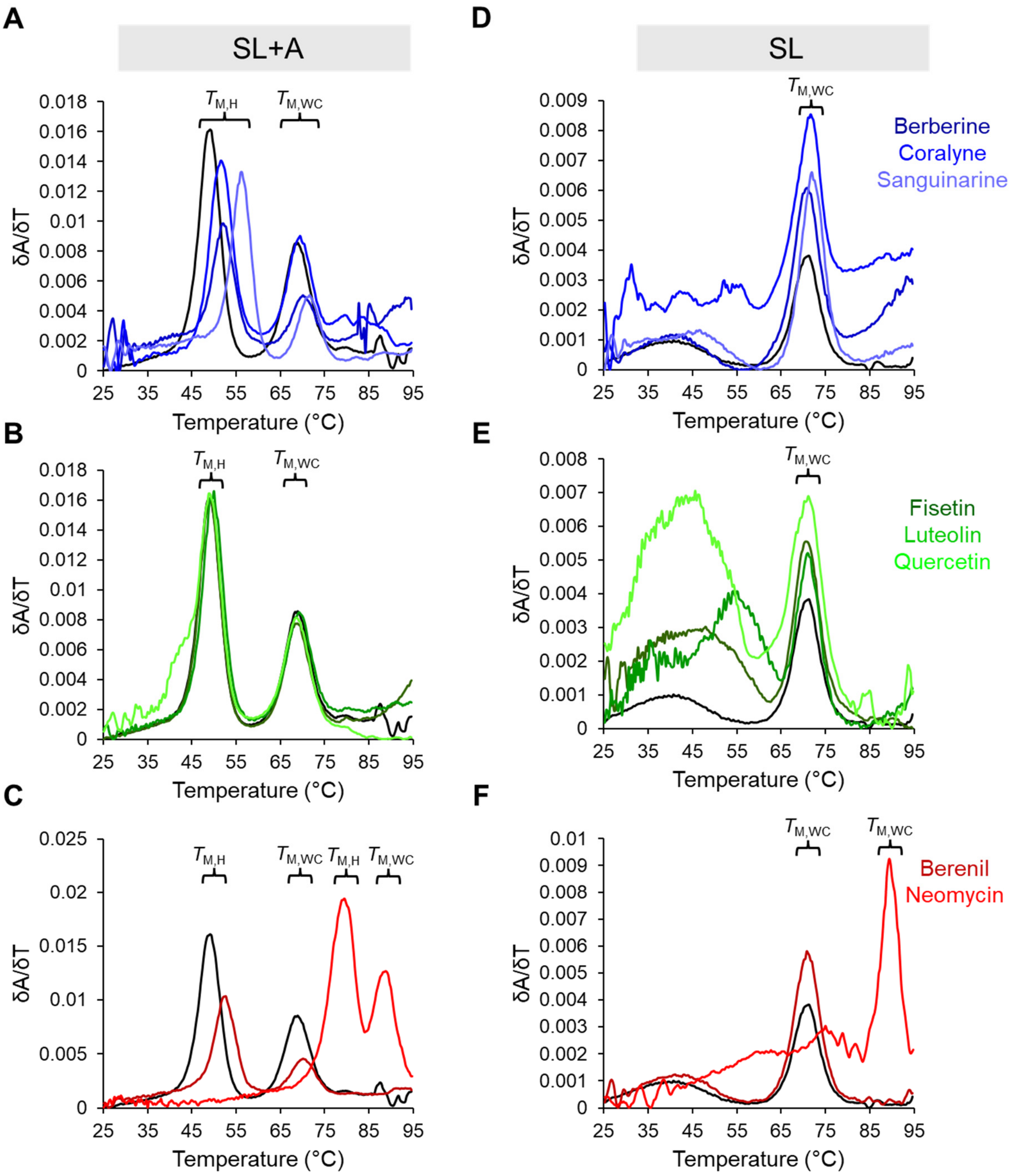

Figure S7 continues onto the next page

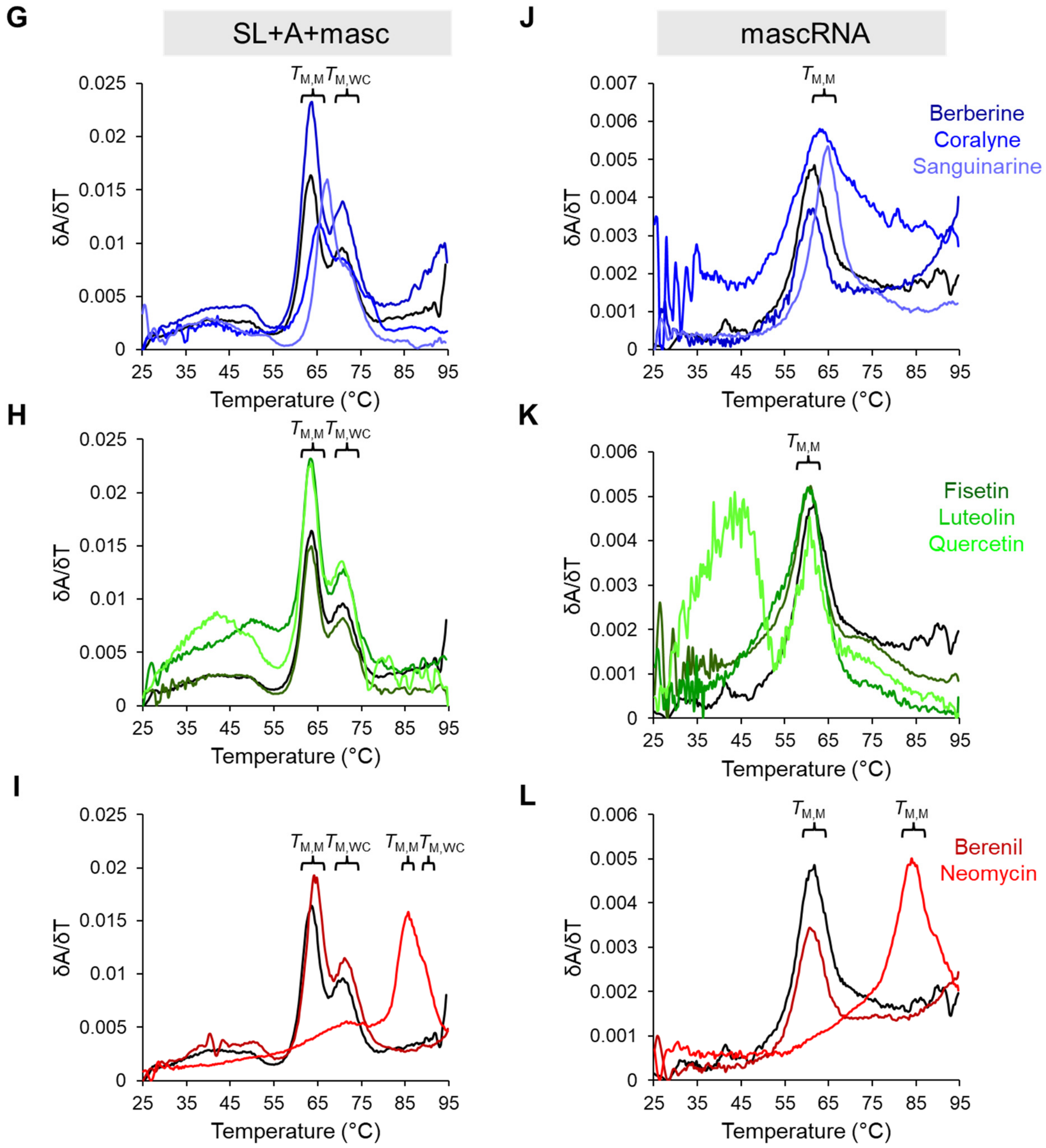

Figure S7. UV thermal melting results for the MALAT1 RNA in the absence and presence of TBMs. Plots of first derivatives of absorbance with respect to temperature versus temperature for the (A-C) SL+A, (D-F) SL, (G-I) SL+A+masc, and (J-L) mascRNA in the absence (black line) and presence of berberine, coralyne, sanguinarine, fisetin, luteolin, quercetin, berenil, and neomycin. The brackets denote three distinct peaks: the Hoogsteen ($T_{M,H}$), mascRNA ($T_{M,M}$) and the Watson-Crick ($T_{M,WC}$) melting temperatures. All colors are as defined in the legend of Figure 1D. This figure corresponds to **Figure 2, 4,** and **Table 1.** Raw and processed data are presented in **File S1**.

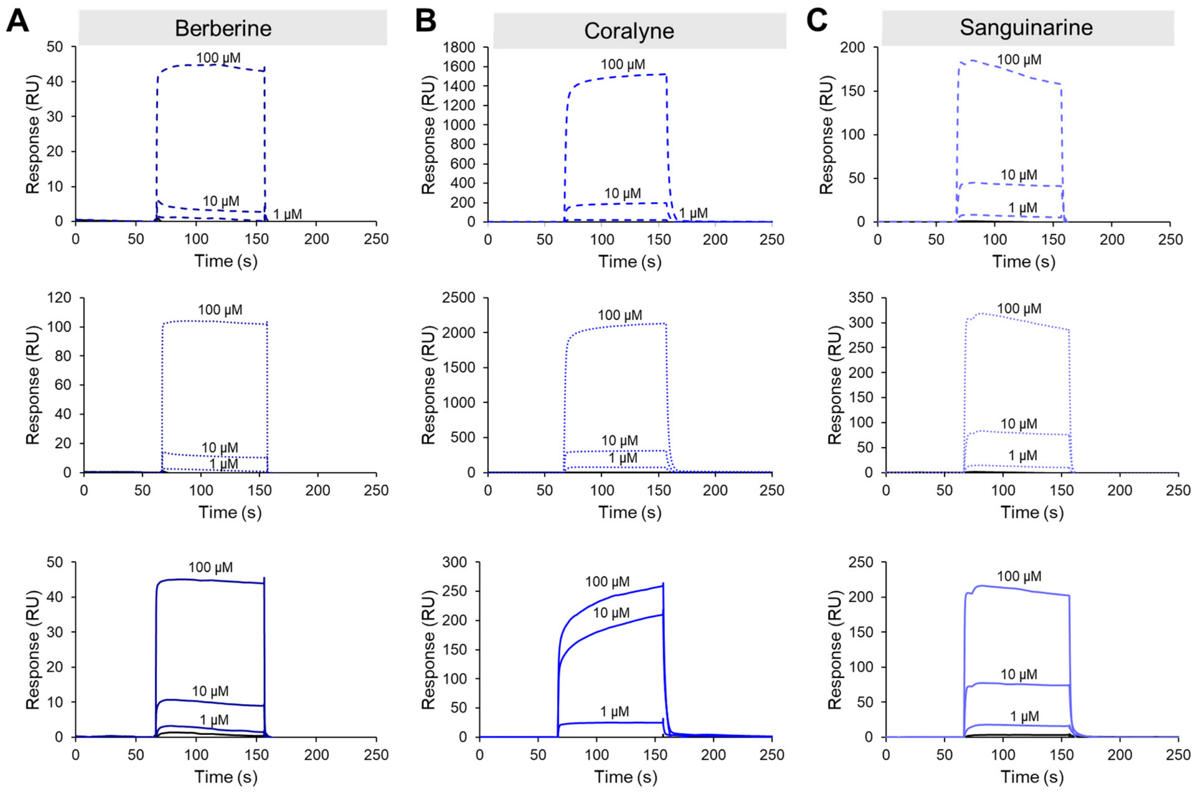

Figure S8 continues onto the next page.

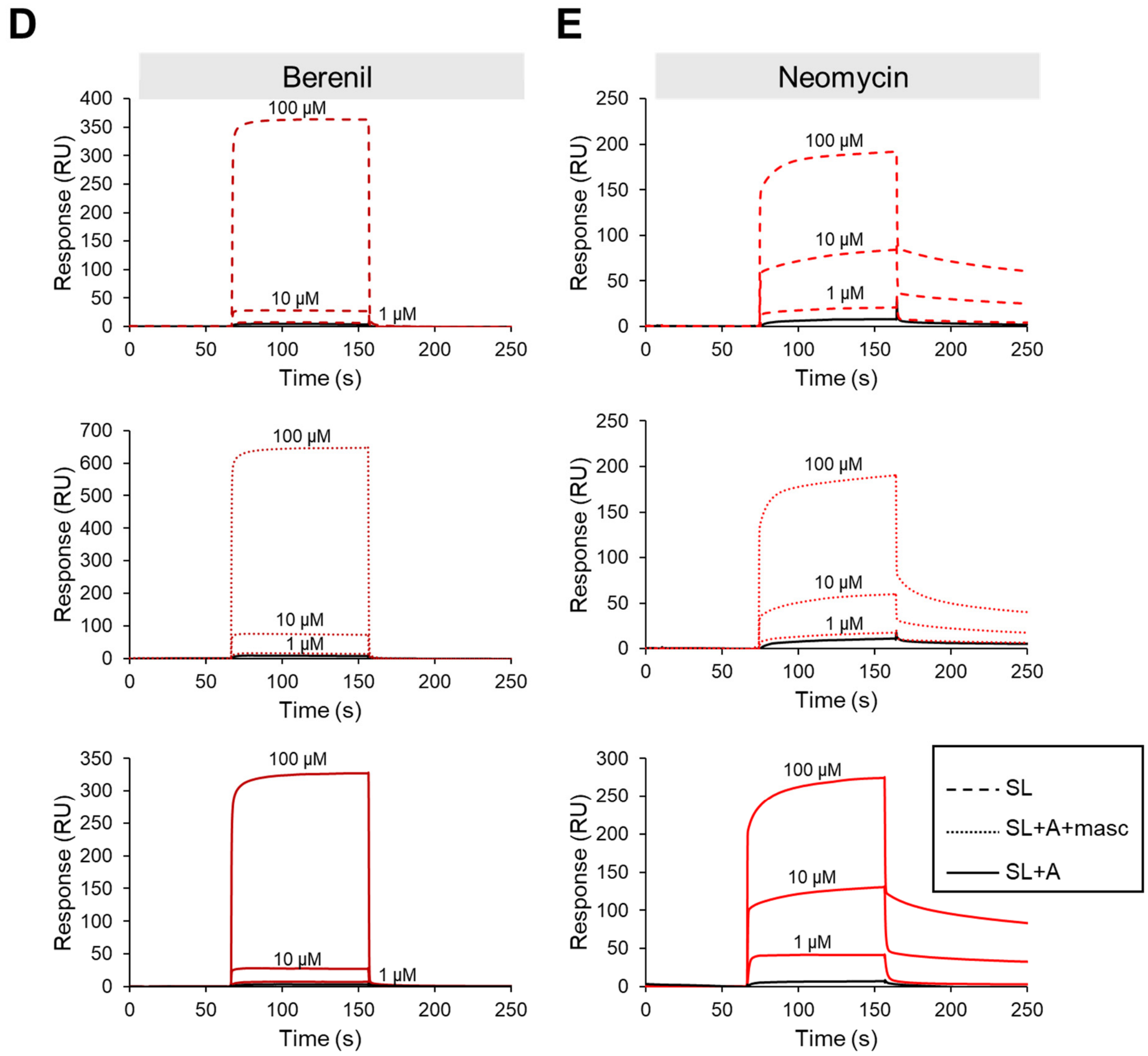

Figure S8. SPR sensogram plots comparing how each TBM binds to the three different RNAs at all concentrations. Representative SPR sensograms are shown for (A) berberine, (B) coralyne, (C) sanguinarine, (D) berenil, and (E) neomycin. Dashed line represents MALAT1 SL, dotted line represents SL+A+masc, and solid line represents SL+A. This figure corresponds to **Figure 5**. Raw sensogram data are presented in **File S3**.

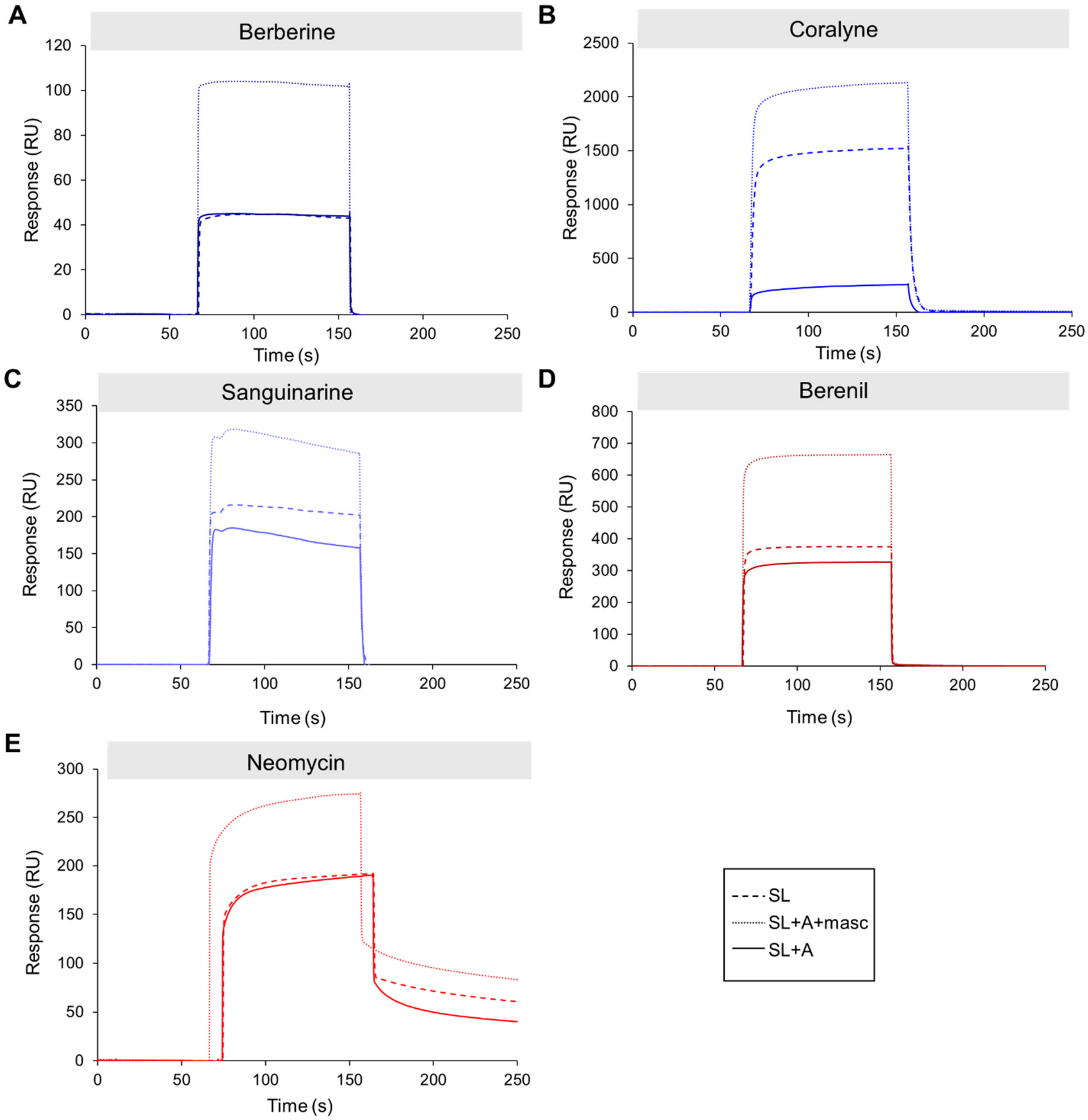

Figure S9. SPR sensogram plots comparing how each TBM interacts with the three different RNAs. SPR sensograms for all three MALAT1 RNAs in the presence of 100 µM of (A) berberine, (B) coralyne, (C) sanguinarine, (D) berenil, and (E) neomycin. Dashed line represents MALAT1 SL, dotted line represents SL+A+masc, and solid line represents SL+A. This figure corresponds to **Figure 5**. Raw sensogram data are presented in **File S3**.

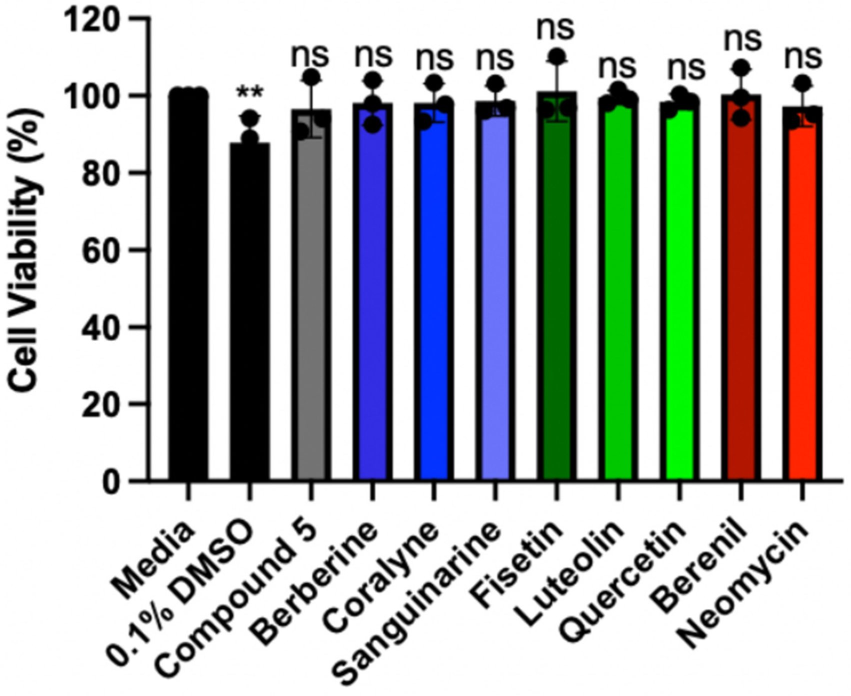

Figure S10. MTT cell viability assay for TBM-treated HCT116 cells. Shown is the percent cell viability after 0.1% DMSO treatment or 1 μM TBM treatment. The percent represents an average ± standard deviation over biological replicates (n=3). Raw and processed data are presented in **File S4**.

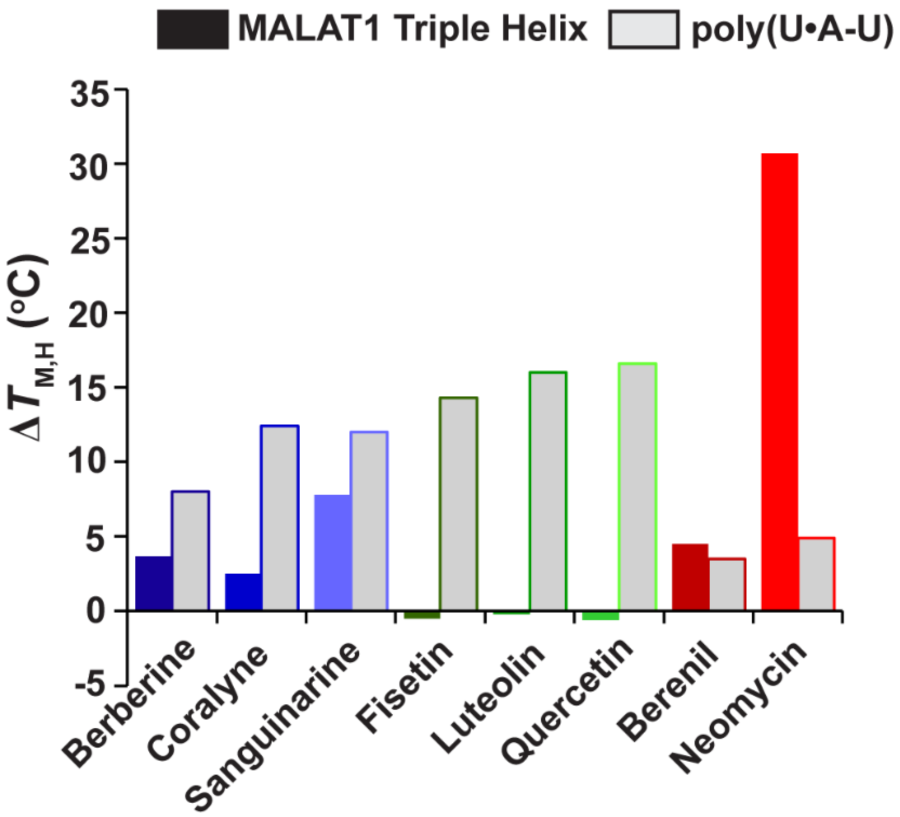

Figure S11. Bar plot showing $\Delta T_{M,H}$ values for the MALAT1 triple helix (solid color) and the poly(U•A-U) triple helix (light gray) in the presence of TBMs. The colors are as defined in the legend of **Figure 1D**. For the MALAT1 triple helix, TBM:RNA molar ratios were 20:1. For the poly(U•A-U) triple helix, the reported molar ratio was at 0.1:1 for alkaloids [10–12], 0.5:1 for flavonoids [13–15] and berenil [16] and 0.025:1 for neomycin [17].

SUPPLEMENTARY TABLES

Table S1. T_M values for the MALAT1 triple helix variants a-c in the absence or presence of TBMs.

TBM	MALAT1 Triple Helix Variants					
	a		b		c	
	$T_{M,H}$	$T_{M,WC}$	$T_{M,H}$	$T_{M,WC}$	$T_{M,H}$	$T_{M,WC}$
**0.1% DMSO**	61.1 ± 0.4	70.8 ± 0.4	54.5 ± 0.4	71.7 ± 0.2	50.2 ± 0.5	71.9 ± 0.7
**Berberine**	61.7 ± 0.4	73.6 ± 0.8	54.9 ± 0.8	71.8 ± 0.6	50.1 ± 0.4	71.1 ± 0.5
**Coralyne**	60.3 ± 0.2	69.9 ± 0.6	52.0 ± 0.1	69.3 ± 0.2	48.6 ± 0.2	69.8 ± 0.1
**Sanguinarine**	63.8 ± 0.1	72.7 ± 0.6	57.6 ± 0.5	73.3 ± 0.7	55.1 ± 0.1	73.1 ± 0.7
**Fisetin**	56.5 ± 0.4	68.8 ± 0.4	51.0 ± 0.2	68.8 ± 0.1	46.3 ± 0.6	67.8 ± 1.0
**Luteolin**	55.9 ± 0.8	69.0 ± 0.2	50.7 ± 0.2	69.0 ± 0.2	46.7 ± 0.4	68.8 ± 0.1
**Quercetin**	56.2 ± 0.5	68.7 ± 0.4	50.7 ± 0.2	68.6 ± 0.4	46.5 ± 0.7	68.7 ± 0.2
**Berenil**	61.4 ± 0.8	71.0 ± 0.4	53.7 ± 0.2	70.7 ± 0.3	51.0 ± 0.9	71.6 ± 0.5
**Neomycin**	82.7 ± 1.0	88.8 ± 0.0	65.6 ± 0.4	76.9 ± 0.5	82.1 ± 0.4	89.9 ± 0.1

The T_M values are the average ± standard deviation of three independent melting experiments.

Table S2. ΔT_M values for the MALAT1 triple helix variants a-c in the absence or presence of TBMs.

TBM	MALAT1 Triple Helix Variants					
	a		b		c	
	$\Delta T_{M,H}$	$\Delta T_{M,WC}$	$\Delta T_{M,H}$	$\Delta T_{M,WC}$	$\Delta T_{M,H}$	$\Delta T_{M,WC}$
**0.1% DMSO**	-	-	-	-	-	-
**Berberine**	0.6	2.8	0.4	0.1	-0.1	-0.8
**Coralyne**	-0.8	-0.9	-2.5	-2.4	-1.6	-2.1
**Sanguinarine**	2.7	1.9	3.1	1.6	4.9	1.2
**Fisetin**	-4.6	-2.0	-3.5	-2.9	-3.9	-4.1
**Luteolin**	-5.2	-1.8	-3.8	-2.7	-3.5	-3.1
**Quercetin**	-4.9	-2.1	-3.8	-3.1	-3.7	-3.2
**Berenil**	0.3	0.2	-0.8	-1.0	0.8	-0.3
**Neomycin**	21.6	18	11.1	5.2	31.9	18

ΔT_M values were calculated as $T_{M(RNA+TBM)}$ - $T_{M(RNA+DMSO)}$.

Table S3. T_M values for the MALAT1 triple helix variants d-g in the absence or presence of TBMs.

TBM	MALAT1 Triple Helix Variants							
	d		e		f		g	
	$T_{M,H}$	$T_{M,WC}$	$T_{M,H}$	$T_{M,WC}$	$T_{M,H}$	$T_{M,WC}$	$T_{M,H}$	$T_{M,WC}$
**0.1% DMSO**	54.6 ± 0.2	71.4 ± 0.5	52.5 ± 0.6	70.6 ± 0.6	51.8 ± 0.6	71.2 ± 0.7	47.3 ± 0.5	71.9 ± 0.5
**Berberine**	54.1 ± 0.1	70.8 ± 0.2	52.3 ± 0.6	71.1 ± 0.2	50.5 ± 0.1	70.0 ± 0.6	47.9 ± 0.3	70.8 ± 0.2
**Coralyne**	56.0 ± 0.5	69.9 ± 0.6	47.5 ± 1.0	71.2 ± 0.1	51.3 ± 0.6	69.6 ± 0.0	47.3 ± 0.3	69.6 ± 0.0
**Sanguinarine**	56.6 ± 0.1	72.1 ± 0.1	53.6 ± 0.9	73.2 ± 0.5	57.3 ± 0.2	72.8 ± 0.4	53.4 ± 0.4	72.0 ± 0.0
**Fisetin**	53.4 ± 0.0	70.6 ± 0.4	52.2 ± 0.2	70.5 ± 0.2	48.8 ± 0.5	68.5 ± 0.5	47.5 ± 0.1	70.9 ± 0.1
**Luteolin**	55.8 ± 0.8	70.5 ± 0.4	52.4 ± 0.8	70.4 ± 0.4	48.9 ± 0.6	68.8 ± 0.4	44.7 ± 0.7	68.5 ± 0.5
**Quercetin**	45.3 ± 0.8	70.5 ± 0.6	44.0 ± 1.0	68.0 ± 0.3	46.4 ± 1.0	68.5 ± 0.1	44.5 ± 0.8	68.7 ± 0.2
**Berenil**	54.8 ± 0.5	70.9 ± 0.6	51.4 ± 0.9	70.1 ± 0.4	52.1 ± 0.3	70.6 ± 0.4	48.1 ± 0.4	70.9 ± 0.4
**Neomycin**	81.4 ± 0.1	89.2 ± 0.4	80.6 ± 0.4	89.5 ± 0.6	78.2 ± 0.1	89.4 ± 0.2	63.2 ± 1.0	77.3 ± 0.7

The T_M values are the average ± standard deviation of three independent melting experiments.

Table S4. ΔT_M values for the MALAT1 triple helix variants d-g in the absence or presence of TBMs.

TBM	MALAT1 Triple Helix Variants							
	d		e		f		g	
	$\Delta T_{M,H}$	$\Delta T_{M,WC}$	$\Delta T_{M,H}$	$\Delta T_{M,WC}$	$\Delta T_{M,H}$	$\Delta T_{M,WC}$	$\Delta T_{M,H}$	$\Delta T_{M,WC}$
**0.1% DMSO**	-	-	-	-	-	-	-	-
**Berberine**	-0.5	-0.6	-0.2	0.5	-1.3	-1.2	0.6	-1.1
**Coralyne**	1.4	-1.5	-5.0	0.6	-0.5	-1.6	0.0	-2.3
**Sanguinarine**	2.0	0.7	1.1	2.6	5.5	1.6	6.1	0.1
**Fisetin**	-1.2	-0.8	-0.3	-0.1	-3.0	-2.7	0.2	-1.0
**Luteolin**	1.2	-0.9	-0.1	-0.2	-2.9	-2.4	-2.6	-3.4
**Quercetin**	-9.3	-0.9	-8.5	-2.6	-5.4	-2.7	-2.8	-3.2
**Berenil**	0.2	-0.5	-1.1	-0.5	0.3	-0.6	0.8	-1.0
**Neomycin**	26.8	17.8	28.1	18.9	26.4	18.2	15.9	5.4

ΔT_M values were calculated as $T_{M(RNA+TBM)}$ - $T_{M(RNA+DMSO)}$.

Table S5. Summary of characteristics for each TBM and its subcellular localization.

TBM	Pharmacological Effects	Biological Molecules Impacted by TBM	Cellular Localization
Berberine	Antibacterial [18], anticancer [19] , anti-inflammatory [18], antioxidant [18], hypoglycemic activities [18]	d(CGTACG)$_2$DNA [18], Aβ1-42 [18], ABL1 [20], AKR1B10 [21], BACE1 [18], BmrR [18], Ftsz [18], JAK2 [18], MD-2 [18], MET [18], NEK7 [18], PLA2 [18], PSMα2 [18], QacR [18], RamR [18], RXRα [22], TIGAR [23], TGHBR1 [18]	Mitochondria in K1735-M2 mouse melanoma cells, WM793 human melanoma cells, and human breast carcinoma MCF7 cells [24,25]
Coralyne	Anticancer [26], anti-inflammatory [27], antimicrobial [10]	DNA duplexes [28], DNA T•A-T triplexes [28], RNA triplexes [11], MMP9 [29], Topoisomerase I [30]	Cytoplasm in human lung carcinoma A549 cells [27]
Sanguinarine	Anticancer [31], antifungal [32], anti-inflammatory [32], antimicrobial [32]	H-DNA triplexes [33], telomeres [33], telomeric G-quadruplexes [34], dsRNA [33], mRNA poly(A) [33], tRNA [33], Aurora Kinase a [35], cyclin-dependent kinase 2 [35], histones [33]	Nucleus in human breast carcinoma MCF7 cells [36]
Fisetin	Anticancer [37], anti-inflammation [37], neuroprotection [37]	dsDNA [38], G4-DNA [39], U-A RNA duplexes [13], U•A-U RNA triplexes [13], β-tubulin [40], reduces RNA pol I activity [41], SUMO [42]	Nucleolus in breast cancer SUM159 cells [41]
Luteolin	Anti-allergy [43], anticancer [43,44], anti-inflammation [43,44], antioxidant [43,44]	dsDNA [45], Msi1 [46], SARS-CoV-2 RNA-dependent RNA polymerase [47], topoisomerase I	Not established

		[45], topoisomerase II [48]	
Quercetin	Anticancer [49], anti-diabetic [50], anti-inflammatory [50], antimicrobial [50], antioxidant [50]	MALAT1 [51], Bcl-2 [52], hnRNPA1 [53], JAK2 [54], MAPK/ERK1/2 [55], MMP-1 [56], NS5A [57], P13K/Akt/mTOR [55], PKCδ [54], VEGF [55]	Nucleus in human liver cancer HepG2 cells [58]
Berenil	Anticancer [59], anti-inflammatory [60], antiparasitic [60]	G-quadruplexes [61], minor groove AT-rich DNA [62], DNA duplexes [63], RNA-DNA triplexes [64], RNA duplexes [63], ACE II [60], DNA topoisomerase II [65]	Not established
Neomycin	Antibacterial [66], antiviral [67]	G-quadruplexes [68], Triplex DNA [17], DNA:RNA hybrid duplex [69], 16s ribosomal RNA [70], ssRNA [66], RNA triplexes [17], G-protein-coupled receptors [71], PIP2 [72], Rev-response element (RRE) [73], TAR [73,74]	Nucleoli when conjugated to thiazole orange in mouse reticulum cell sarcoma J774A.1 cells and human breast carcinoma MCF7 cells [75]

**Table S6.** *In vitro* transcribed RNAs used in this study.	
MALAT1 triple helix (SL+A)	5′-GGAAGGUUUUUCUUUUCCUGAGAAAACAACACGUAUUGUUUUCU<br>CAGGUUUUGCUUUUUGGCCUUUUUCUAGCUUAAAAAAAAAAAAAGCAAA<br>A-3′
Variant **a**	5′-GGAAGGU**C**UUUCUU**C**UCCUGAGAAAACAACACGUAUU<br>GUUUUCUCAGGU**C**UUGCUUU**C**UGGCCUUUUUCUAGCUUAA<br>AAAAAAA**G**AAAGCAA**G**A-3′
Variant **b**	5′-GGAAGGUUUUUCUUUUCCUGAGAAAACAACACGUAUU<br>GUUUUCUCAGGUUUUCUUUUUGGCCUUUUUCUAGCUUAA<br>AAAAAAAAAAAGAAAA-3′
Variant **c**	5′-GGAAGGUUUUU**U**UUUUCCUGAGAAAACAACACGUAUU<br>GUUUUCUCAGGUUUU**U**UUUUUGGCCUUUUUCUAGCUUAA<br>AAAAAAAAAAA**A**AAAAA-3′
Variant **d**	5′-GGAAGGUUCUUCCUGAGAAAACAACACGUAUU<br>GUUUUCUCAGGUUCUUGGCCUUUUUCUAGCUUAA<br>AAAAAAAAGAA-3′
Variant **e**	5′-GGAAGGUUUCUUCCUGAGAAAACAACACGUAUU<br>GUUUUCUCAGGUUCUUUGGCCUUUUUCUAGCUUAA<br>AAAAAAAAAGAA-3′
Variant **f**	5′-GGAAGGUUUUUCUUUUCCUGAGAAAACAACACGUAUU<br>GUUUUCUCAGGUUUUGCUUUUUGG**G**CUUUUUCUAGCUUAA<br>AAAAAAAAAAAGCAAAA-3′
Variant **g**	5′-GGAA**C**GUUUUUCUUUUCCUGAGAAAACAACACGUAUU<br>GUUUUCUCAGGUUUUGCUUUUUGGCCUUUUUCUAGCUUAA<br>AAAAAAAAAAAGCAAAA-3′
MALAT1 SL	5′-GGAAGGUUUUUCUUUUCCUGAGAAAACAACACGUAUUGUUUUCU<br>CAGGUUUUGCUUUUUGGCCUUUUU-3′
MALAT1 SL+A+masc	5′-GGAAGGUUUUUCUUUUCCUGAGAAAACAACACGUAUUGUUUUCU<br>CAGGUUUUGCUUUUUGGCCUUUUUCUAGCUUAAAAAAAAAAAAAGCAAA<br>AGAUGCUGGUGGUUGGCACUCCUGGUUUCCAGGACGGGGUUCAAAUCCC<br>UGCGGCGUCUUUGCUUU-3′
mascRNA	5′-GAUGCUGGUGGUUGGCACUCCUGGUUUCCAGGACGGGGUUCAAU<br>CCCUGCGGCGUC-3′
Pre-mascRNA	5′-AAAGCAAAAGAUGCUGGUGGUUGGCACUCCUGGUUUCCAGGACGG<br>GGUUCAAAUCCCUGCGGCGUCUUUGCUUU-3′
Extended MALAT1 SL (SPR assays)	5′-*GGCGGAACUUAGCCACUGUGAAAU*GGAAGGUUUUUCUUUUC<br>CUGAGAAAACAACACGUAUUGUUUUCUCAGGUUUUGCUUUUUGGCCUU<br>UUU-3′
Extended MALAT1 SL+A+masc (SPR assays)	5′-*GGCGGAACUUAGCCACUGUGAAAU*GGAAGGUUUUUCUUUUCC<br>UGAGAAAACAACACGUAUUGUUUUCUCAGGUUUUGCUUUUUGGCCUUUU<br>UCUAGCUUAAAAAAAAAAAAAGCAAAAGAUGCUGGUGGUUGGCACUCCU<br>GGUUUCCAGGACGGGGUUCAAAUCCCUGCGGCGUCUUUGCUUU-3′
Extended MALAT1 SL+A (SPR assays)	5′-*GGCGGAACUUAGCCACUGUGAAAU*GGAAGGUUUUUCUUUUCC<br>UGAGAAAACAACACGUAUUGUUUUCUCAGGUUUUGCUUUUUGGCCUUUU<br>UCUAGCUUAAAAAAAAAAAAAGCAAAA-3′

Nucleotides highlighted in blue represent the Hoogsteen strand, purple represent the Watson strand and green represent the Crick strand of the major-groove triple helix. Bold letters represent mutated nucleotides. In the extended MALAT1 triple helix for SPR, the 5′ extension of 24 nucleotides is indicated in italics.

Table S7. Average T_M values for the MALAT1 processing RNA in the absence or presence of TBMs.

TBM	SL+A		SL	SL+A+masc			mascRNA
	$T_{M,H}$	$T_{M,WC}$	$T_{M,WC}$	$T_{M,H}$	$T_{M,M}$	$T_{M,WC}$	$T_{M,M}$
**0.1% DMSO**	49.6 ± 0.4	69.0 ± 0.0	71.2 ± 0.3	48.5 ± 0.9	64.0 ± 0.3	70.1 ± 0.5	61.6 ± 0.1
**Berberine**	53.3 ± 0.8	70.8 ± 0.4	70.7 ± 0.1	48.4 ± 0.1	63.8 ± 0.1	70.6 ± 0.3	60.8 ± 0.3
**Coralyne**	52.1 ± 0.5	70.0 ± 0.3	72.2 ± 0.9	ND	65.7 ± 0.0	70.6 ± 0.5	63.8 ± 0.7
**Sanguinarine**	57.4 ± 0.7	72.2 ± 0.4	72.3 ± 0.4	50.7 ± 0.5	67.5 ± 0.0	71.8 ± 0.6	64.8 ± 0.2
**Fisetin**	49.1 ± 0.1	69.0 ± 0.2	70.8 ± 0.2	48.7 ± 0.5	63.5 ± 0.1	70.7 ± 0.1	60.9 ± 0.0
**Luteolin**	49.4 ± 0.5	68.8 ± 0.4	71.0 ± 0.1	49.1 ± 0.5	63.4 ± 0.3	71.2 ± 0.1	60.1 ± 0.1
**Quercetin**	49.0 ± 0.1	68.9 ± 0.3	70.9 ± 0.1	ND	64.1 ± 0.4	71.2 ± 0.8	60.8 ± 0.1
**Berenil**	54.1 ± 0.9	70.8 ± 0.4	70.9 ± 0.1	50.4 ± 0.0	64.0 ± 0.1	70.7 ± 0.3	60.7 ± 0.1
**Neomycin**	80.3 ± 0.6	89.2 ± 0.4	89.9 ± 0.3	72.3 ± 0.5	86.2 ± 0.4	96.1 ± 0.4	83.9 ± 0.1

The T_M values are the average ± standard deviation of three independent melting experiments.
ND indicates no detectable melting peak or peak height is less than 15% compared to peak height corresponding to $T_{m,WC}$.

Table S8. ΔT_M values for the MALAT1 processing RNA in the absence or presence of TBMs.

TBM	SL+A		SL	SL+A+masc			mascRNA
	$\Delta T_{M,H}$	$\Delta T_{M,WC}$	$\Delta T_{M,WC}$	$\Delta T_{M,H}$	$\Delta T_{M,M}$	$\Delta T_{M,WC}$	$\Delta T_{M,M}$
**0.1% DMSO**	-	-	-	-	-	-	-
**Berberine**	3.7	1.8	-0.5	-0.1	-0.2	0.5	-0.8
**Coralyne**	2.5	0.8	1.0	ND	1.7	0.5	2.2
**Sanguinarine**	7.8	3.2	1.1	2.2	3.5	1.7	3.2
**Fisetin**	-0.5	0.0	-0.4	0.2	-0.5	0.6	-0.7
**Luteolin**	-0.2	-0.2	-0.2	0.6	-0.6	1.1	-1.5
**Quercetin**	-0.6	-0.1	-0.3	ND	-0.4	0.6	-0.8
**Berenil**	4.5	1.8	-0.3	1.9	0.0	0.6	-0.9
**Neomycin**	30.7	20.2	18.7	23.8	22.2	26.0	22.3

The T_M values are the average ± standard deviation of three independent melting experiments.
ND indicates no detectable melting peak or peak height is less than 15% compared to peak height corresponding to $T_{m,WC}$.

Table S9. Sequences of primers used for RT-qPCR experiments.

Housekeeping genes		
18s rRNA [76]	Forward:<br>Reverse:	5′-CCCAGTAAGTGCGGGTCATAA-3′<br>5′-GATCCGAGGGCCTCACTAA-3′
β-Actin [77]	Forward:<br>Reverse:	5′-CTGGAACGGTGAAGGTGACA-3′<br>5′-AAGGGACTTCCTGTAACAACGCA-3′
GAPDH [78]	Forward:<br>Reverse:	5′-GATCATCAGCAATGCCTCCT-3′<br>5′-TGTGGTCATGAGTCCTTCCA-3′
U6 snRNA [79]	Forward:<br>Reverse:	5′-CTCGCTTCGGCAGCACA-3′<br>5′-AACGCTTCACGAATTTGCGT-3′
Target genes		
Premature-only MALAT1	Forward:<br>Reverse:	5′-CTCTTCAGTAGGGTCATGAAGG-3′<br>5′-GTAGTCAAAGCAAAGACGCCG-3′
MALAT1 [80]	Forward:<br>Reverse:	5′-GATCTAGCACAGACCCTTCAC-3′<br>5′-CGACACCATCGTTACCTTGA-3′
MENβ [80]	Forward:<br>Reverse:	5′-GTGTCCACAGGTCTTAGATTCC-3′<br>5′-TCTGTGTAGTAGGGTGGGATAG-3′
TUG1 (Unspliced Intron 1) [81]	Forward:<br>Reverse:	5′-AAGGCATTGGAAGAGGAAGAG-3′<br>5′-CTGGCTTAGGCAAAGACAAATG-3′

Table S10. Average $2^{-\Delta\Delta CT}$ values and standard deviation for all lncRNA targets.

TBM	**Premature-only MALAT1**	**MALAT1**	**MENβ**	**TUG1 (Unspliced Intron 1)**
0.1% DMSO	1.0 ± 0.0	1.0 ± 0.0	1.0 ± 0.0	1.0 ± 0.0
Compound 5	0.6 ± 0.3	0.4 ± 0.1	1.0 ± 0.4	0.6 ± 0.2
Berberine	0.9 ± 0.2	0.8 ± 0.2	1.1 ± 0.9	1.0 ± 0.4
Coralyne	0.8 ± 0.3	0.8 ± 0.1	0.9 ± 0.2	0.8 ± 0.1
Sanguinarine	0.9 ± 0.3	0.9 ± 0.0	1.0 ± 0.5	0.8 ± 0.1
Fisetin	0.7 ± 0.2	0.7 ± 0.2	1.2 ± 0.4	0.7 ± 0.1
Luteolin	0.6 ± 0.2	0.5 ± 0.0	1.4 ± 0.4	0.6 ± 0.1
Quercetin	0.5 ± 0.1	0.4 ± 0.0	0.8 ± 0.2	0.6 ± 0.1
Berenil	0.6 ± 0.2	0.4 ± 0.0	1.0 ± 0.2	0.7 ± 0.2
Neomycin	0.6 ± 0.3	0.3 ± 0.1	0.7 ± 0.2	0.6 ± 0.2
Average change in expression	0.7 ± 0.1	0.6 ± 0.2	1.0 ± 0.2	0.7 ± 0.1

See **File S4** for raw and processed RT-qPCR data for each replicate.

Table S11. Summary of lncRNA expression levels and cellular localization in HCT116 cells.

Human lncRNA	RNA Expression (TPM)[a]	Cellular Localization
MALAT1	207 [82]	Nuclear Speckles (nucleus) [83,84]
MENβ	93 [82]	Paraspeckles (nucleus) [85–87]
TUG1 (unspliced intron 1)	75 [82]	Predominately in nucleus [88]

[a]TPM values were collected from Expression Atlas, which has data deposited from reference [82].

Table S12. Selectivity factor of each TBM for reducing MALAT1 over MENβ.

TBM	Selectivity Factor[a]
Compound 5 in MMTV-PyMT tumor organoids [5]	~2.0[b]
Compound 5 (this study)	2.2
Compound 16 in MMTV-PyMT tumor organoids [5]	~2.1[b]
Berberine (this study)	1.0
Coralyne (this study)	1.1
Sanguinarine (this study)	1.0
Fisetin (this study)	1.7
Luteolin (this study)	2.6
Quercetin in MCF7 breast cancer cells [51]	~2.0[b]
Quercetin (this study)	1.9
Berenil (this study)	2.3
Neomycin (this study)	2.1

[a]Selectivity factor was calculated as (MALAT1 fold change/MENβ fold change). Values used for MALAT1 and MENβ are those that correspond to **Figure 6B-C**.
[b]Fold change values were estimated based on visual inspection of RT-qPCR plots because $2^{-\Delta\Delta CT}$ values were not published.

References

1. Abramson, J.; Adler, J.; Dunger, J.; Evans, R.; Green, T.; Pritzel, A.; Ronneberger, O.; Willmore, L.; Ballard, A.J.; Bambrick, J.; et al. Accurate Structure Prediction of Biomolecular Interactions with AlphaFold 3. *Nature* **2024**, *630*, 493–500, doi:10.1038/s41586-024-07487-w.
2. Brown, J.A.; Bulkley, D.; Wang, J.; Valenstein, M.L.; Yario, T.A.; Steitz, T.A.; Steitz, J.A. Structural Insights into the Stabilization of MALAT1 Noncoding RNA by a Bipartite Triple Helix. *Nat. Struct. Mol. Biol.* **2014**, *21*, 633–640, doi:10.1038/nsmb.2844.
3. Veenbaas, S.D.; Felder, S.; Weeks, K.M. fpocketR: A Platform for Identification and Analysis of Ligand-Binding Pockets in RNA. *bioRxiv* **2025**, 2025.03.25.645323, doi:10.1101/2025.03.25.645323.
4. Donlic, A.; Morgan, B.S.; Xu, J.L.; Liu, A.; Roble, C.; Hargrove, A.E. Discovery of Small Molecule Ligands for MALAT1 by Tuning an RNA-Binding Scaffold. *Angew. Chem. Int. Ed Engl.* **2018**, *57*, 13242–13247, doi:10.1002/anie.201808823.
5. Abulwerdi, F.A.; Xu, W.; Ageeli, A.A.; Yonkunas, M.J.; Arun, G.; Nam, H.; Schneekloth, J.S.Jr.; Dayie, T.K.; Spector, D.; Baird, N.; et al. Selective Small-Molecule Targeting of a Triple Helix Encoded by the Long Noncoding RNA, MALAT1. *ACS Chem. Biol.* **2019**, *14*, 223–235, doi:10.1021/acschembio.8b00807.
6. Donlic, A.; Zafferani, M.; Padroni, G.; Puri, M.; Hargrove, A.E. Regulation of MALAT1 Triple Helix Stability and in Vitro Degradation by Diphenylfurans. *Nucleic Acids Res.* **2020**, *48*, 7653–7664, doi:10.1093/nar/gkaa585.
7. François-Moutal, L.; Miranda, V.G.; Mollasalehi, N.; Gokhale, V.; Khanna, M. In Silico Targeting of the Long Noncoding RNA MALAT1. *ACS Med. Chem. Lett.* **2021**, *12*, 915–921, doi:10.1021/acsmedchemlett.1c00060.
8. Zafferani, M.; Martyr, J.G.; Muralidharan, D.; Montalvan, N.I.; Cai, Z.; Hargrove, A.E. Multiassay Profiling of a Focused Small Molecule Library Reveals Predictive Bidirectional Modulation of the lncRNA MALAT1 Triplex Stability In Vitro. *ACS Chem. Biol.* **2022**, *17*, 2437–2447, doi:10.1021/acschembio.2c00124.
9. Pernak, M.; Fleurisson, C.; Delorme, C.; Moumné, R.; Benedetti, E.; Micouin, L.; Azoulay, S.; Foricher, Y.; Duca, M. Development of Comprehensive Screening and Assessment Assays for Small-Molecule Ligands of MALAT1 lncRNA. *ACS Chem. Biol.* **2025**, *20*, 1068–1076, doi:10.1021/acschembio.5c00061.
10. Das, S.; Kumar, G.S.; Ray, A.; Maiti, M. Spectroscopic and Thermodynamic Studies on the Binding of Sanguinarine and Berberine to Triple and Double Helical DNA and RNA Structures. *J. Biomol. Struct. Dyn.* **2003**, *20*, 703–713, doi:10.1080/07391102.2003.10506887.
11. Sinha, R.; Kumar, G.S. Interaction of Isoquinoline Alkaloids with an RNA Triplex: Structural and Thermodynamic Studies of Berberine, Palmatine, and Coralyne Binding to Poly(U).Poly(A)*Poly(U). *J. Phys. Chem. B* **2009**, *113*, 13410–13420, doi:10.1021/jp9069515.
12. Bhowmik, D.; Das, S.; Hossain, M.; Haq, L.; Suresh Kumar, G. Biophysical Characterization of the Strong Stabilization of the RNA Triplex Poly(U)•poly(A)*poly(U) by 9-O-(ω-Amino) Alkyl Ether Berberine Analogs. *PloS One* **2012**, *7*, e37939, doi:10.1371/journal.pone.0037939.
13. Bhuiya, S.; Haque, L.; Goswami, R.; Das, S. Multispectroscopic and Theoretical Exploration of the Comparative Binding Aspects of Bioflavonoid Fisetin with Triple- and Double-Helical Forms of RNA. *J. Phys. Chem. B* **2017**, *121*, 11037–11052, doi:10.1021/acs.jpcb.7b07972.
14. Tiwari, R.; Haque, L.; Bhuiya, S.; Das, S. Third Strand Stabilization of Poly(U)·poly(A)* Poly(U) Triplex by the Naturally Occurring Flavone Luteolin: A Multi-Spectroscopic Approach. *Int. J. Biol. Macromol.* **2017**, *103*, 692–700, doi:10.1016/j.ijbiomac.2017.05.115.

15. Pradhan, A.B.; Bhuiya, S.; Haque, L.; Das, S. Role of Hydroxyl Groups in the B-Ring of Flavonoids in Stabilization of the Hoogsteen Paired Third Strand of Poly(U).Poly(A)*Poly(U) Triplex. *Arch. Biochem. Biophys.* **2018**, *637*, 9–20, doi:10.1016/j.abb.2017.11.008.
16. Pilch, D.S.; Kirolos, M.A.; Breslauer, K.J. Berenil Binding to Higher Ordered Nucleic Acid Structures: Complexation with a DNA and RNA Triple Helix. *Biochemistry* **1995**, *34*, 16107–16124, doi:10.1021/bi00049a026.
17. Arya, D.P.; Coffee, R.L.; Willis, B.; Abramovitch, A.I. Aminoglycoside–Nucleic Acid Interactions: Remarkable Stabilization of DNA and RNA Triple Helices by Neomycin. *J. Am. Chem. Soc.* **2001**, *123*, 5385–5395, doi:10.1021/ja003052x.
18. Sun, P.; Wang, Z.; Ma, Y.; Liu, Y.; Xue, Y.; Li, Y.; Gao, X.; Wang, Y.; Chu, M. Advance in Identified Targets of Berberine. *Front. Pharmacol.* **2025**, *16*, doi:10.3389/fphar.2025.1500511.
19. La, X.; Zhang, L.; Li, Z.; Yang, P.; Wang, Y. Berberine-Induced Autophagic Cell Death by Elevating GRP78 Levels in Cancer Cells. *Oncotarget* **2017**, *8*, 20909–20924, doi:10.18632/oncotarget.14959.
20. Yin, Z.; Huang, G.; Gu, C.; Liu, Y.; Yang, J.; Fei, J. Discovery of Berberine That Targetedly Induces Autophagic Degradation of Both BCR-ABL and BCR-ABL T315I through Recruiting LRSAM1 for Overcoming Imatinib Resistance. *Clin. Cancer Res.* **2020**, *26*, 4040–4053, doi:10.1158/1078-0432.CCR-19-2460.
21. Yang, S.; Cao, S.-J.; Li, C.-Y.; Zhang, Q.; Zhang, B.-L.; Qiu, F.; Kang, N. Berberine Directly Targets AKR1B10 Protein to Modulate Lipid and Glucose Metabolism Disorders in NAFLD. *J. Ethnopharmacol.* **2024**, *332*, 118354, doi:10.1016/j.jep.2024.118354.
22. Ruan, H.; Zhan, Y.Y.; Hou, J.; Xu, B.; Chen, B.; Tian, Y.; Wu, D.; Zhao, Y.; Zhang, Y.; Chen, X.; et al. Berberine Binds RXRα to Suppress β-Catenin Signaling in Colon Cancer Cells. *Oncogene* **2017**, *36*, 6906–6918, doi:10.1038/onc.2017.296.
23. Qi, F.; Zhang, M.; Yang, G.; Wang, W.; Hu, Y.; Shen, Y.; Wan, J.; Li, J.; Liu, G.; Deng, Y. Identification of TIGAR, a Direct Proteomic Target Associated with the Hypoglycemic Effect of Berberine. *Fitoterapia* **2025**, *180*, 106332, doi:10.1016/j.fitote.2024.106332.
24. Serafim, T.L.; Oliveira, P.J.; Sardao, V.A.; Perkins, E.; Parke, D.; Holy, J. Different Concentrations of Berberine Result in Distinct Cellular Localization Patterns and Cell Cycle Effects in a Melanoma Cell Line. *Cancer Chemother. Pharmacol.* **2008**, *61*, 1007–1018, doi:10.1007/s00280-007-0558-9.
25. Jin, M.; Ji, X.; Stoika, R.; Liu, K.; Wang, L.; Song, Y. Synthesis of a Novel Fluorescent Berberine Derivative Convenient for Its Subcellular Localization Study. *Bioorganic Chem.* **2020**, *101*, 104021, doi:10.1016/j.bioorg.2020.104021.
26. Bhattacharyya, R.; Gupta, P.; Bandyopadhyay, S.K.; Patro, B.S.; Chattopadhyay, S. Coralyne, a Protoberberine Alkaloid, Causes Robust Photosenstization of Cancer Cells through ATR-P38 MAPK-BAX and JAK2-STAT1-BAX Pathways. *Chem. Biol. Interact.* **2018**, *285*, 27–39, doi:10.1016/j.cbi.2018.02.032.
27. Węgierek-Ciuk, A.; Arabski, M.; Ciepluch, K.; Brzóska, K.; Lisowska, H.; Czerwińska, M.; Stępkowski, T.; Lis, K.; Lankoff, A. Coralyne Radiosensitizes A549 Cells by Upregulation of CDKN1A Expression to Attenuate Radiation Induced G2/M Block of the Cell Cycle. *Int. J. Mol. Sci.* **2021**, *22*, 5791, doi:10.3390/ijms22115791.
28. Basu, A.; Suresh Kumar, G. Coralyne Induced Self-Structure in Polyadenylic Acid: Thermodynamics of the Structural Reorganization. *J. Chem. Thermodyn.* **2016**, *101*, 221–226, doi:10.1016/j.jct.2016.06.002.
29. Vempati, R.K.; Malla, R.R. Coralyne Targets the Catalytic Domain of MMP9: An *In Silico* and *In Vitro* Investigation. *Crit. Rev. Oncog.* **2025**, *30*, doi:10.1615/CritRevOncog.2024056393.
30. Gatto, B.; Sanders, M.M.; Yu, C.; Wu, H.Y.; Makhey, D.; LaVoie, E.J.; Liu, L.F. Identification of Topoisomerase I as the Cytotoxic Target of the Protoberberine Alkaloid Coralyne. *Cancer Res.* **1996**, *56*, 2795–2800.

31. Ahmad, N.; Gupta, S.; Husain, M.M.; Heiskanen, K.M.; Mukhtar, H. Differential Antiproliferative and Apoptotic Response of Sanguinarine for Cancer Cells versus Normal Cells. *Clin. Cancer Res. Off. J. Am. Assoc. Cancer Res.* **2000**, *6*, 1524–1528.
32. Basu, P.; Kumar, G.S. Sanguinarine and Its Role in Chronic Diseases. In *Anti-inflammatory Nutraceuticals and Chronic Diseases*; Gupta, S.C., Prasad, S., Aggarwal, B.B., Eds.; Springer International Publishing: Cham, 2016; pp. 155–172 ISBN 978-3-319-41334-1.
33. Croaker, A.; King, G.J.; Pyne, J.H.; Anoopkumar-Dukie, S.; Liu, L. Sanguinaria Canadensis: Traditional Medicine, Phytochemical Composition, Biological Activities and Current Uses. *Int. J. Mol. Sci.* **2016**, *17*, 1414, doi:10.3390/ijms17091414.
34. Yan, S.; Lin, S.; Chen, K.; Yin, S.; Peng, H.; Cai, N.; Ma, W.; Songyang, Z.; Huang, Y. Natural Product Library Screens Identify Sanguinarine Chloride as a Potent Inhibitor of Telomerase Expression and Activity. *Cells* **2022**, *11*, 1485, doi:10.3390/cells11091485.
35. Li, X.; You, Q. Sanguinarine Identified as a Natural Dual Inhibitor of AURKA and CDK2 through Network Pharmacology and Bioinformatics Approaches. *Sci. Rep.* **2024**, *14*, 29608, doi:10.1038/s41598-024-81063-0.
36. Holy, J.; Lamont, G.; Perkins, E. Disruption of Nucleocytoplasmic Trafficking of Cyclin D1 and Topoisomerase II by Sanguinarine. *BMC Cell Biol.* **2006**, *7*, 13, doi:10.1186/1471-2121-7-13.
37. Zhou, C.; Huang, Y.; Nie, S.; Zhou, S.; Gao, X.; Chen, G. Biological Effects and Mechanisms of Fisetin in Cancer: A Promising Anti-Cancer Agent. *Eur. J. Med. Res.* **2023**, *28*, 297, doi:10.1186/s40001-023-01271-8.
38. Sengupta, B.; Banerjee, A.; Sengupta, P.K. Interactions of the Plant Flavonoid Fisetin with Macromolecular Targets: Insights from Fluorescence Spectroscopic Studies. *J. Photochem. Photobiol. B* **2005**, *80*, 79–86, doi:10.1016/j.jphotobiol.2005.03.005.
39. Bhattacharjee, S.; Chakraborty, S.; Sengupta, P.K.; Bhowmik, S. Exploring the Interactions of the Dietary Plant Flavonoids Fisetin and Naringenin with G-Quadruplex and Duplex DNA, Showing Contrasting Binding Behavior: Spectroscopic and Molecular Modeling Approaches. *J. Phys. Chem. B* **2016**, *120*, 8942–8952, doi:10.1021/acs.jpcb.6b06357.
40. Mukhtar, E.; Adhami, V.M.; Sechi, M.; Mukhtar, H. Dietary Flavonoid Fisetin Binds to β-Tubulin and Disrupts Microtubule Dynamics in Prostate Cancer Cells. *Cancer Lett.* **2015**, *367*, 173–183, doi:10.1016/j.canlet.2015.07.030.
41. Kammerud, S.C.; Metge, B.J.; Elhamamsy, A.R.; Weeks, S.E.; Alsheikh, H.A.; Mattheyses, A.L.; Shevde, L.A.; Samant, R.S. Novel Role of the Dietary Flavonoid Fisetin in Suppressing rRNA Biogenesis. *Lab. Invest.* **2021**, *101*, 1439–1448, doi:10.1038/s41374-021-00642-1.
42. Velazhahan, V.; Glaza, P.; Herrera, A.I.; Prakash, O.; Zolkiewski, M.; Geisbrecht, B.V.; Schrick, K. Dietary Flavonoid Fisetin Binds Human SUMO1 and Blocks Sumoylation of P53. *PLoS ONE* **2020**, *15*, e0234468, doi:10.1371/journal.pone.0234468.
43. Lin, Y.; Shi, R.; Wang, X.; Shen, H.-M. Luteolin, a Flavonoid with Potentials for Cancer Prevention and Therapy. *Curr. Cancer Drug Targets* **2008**, *8*, 634–646.
44. Imran, M.; Rauf, A.; Abu-Izneid, T.; Nadeem, M.; Shariati, M.A.; Khan, I.A.; Imran, A.; Orhan, I.E.; Rizwan, M.; Atif, M.; et al. Luteolin, a Flavonoid, as an Anticancer Agent: A Review. *Biomed. Pharmacother.* **2019**, *112*, 108612, doi:10.1016/j.biopha.2019.108612.
45. Chowdhury, A.R.; Sharma, S.; Mandal, S.; Goswami, A.; Mukhopadhyay, S.; Majumder, H.K. Luteolin, an Emerging Anti-Cancer Flavonoid, Poisons Eukaryotic DNA Topoisomerase I. *Biochem. J.* **2002**, *366*, 653–661, doi:10.1042/BJ20020098.
46. Yi, C.; Li, G.; Ivanov, D.N.; Wang, Z.; Velasco, M.X.; Hernández, G.; Kaundal, S.; Villarreal, J.; Gupta, Y.K.; Qiao, M.; et al. Luteolin Inhibits Musashi1 Binding to RNA and Disrupts Cancer Phenotypes in Glioblastoma Cells. *RNA Biol.* **2018**, *15*, 1420–1432, doi:10.1080/15476286.2018.1539607.

47. Munafò, F.; Donati, E.; Brindani, N.; Ottonello, G.; Armirotti, A.; De Vivo, M. Quercetin and Luteolin Are Single-Digit Micromolar Inhibitors of the SARS-CoV-2 RNA-Dependent RNA Polymerase. *Sci. Rep.* **2022**, *12*, 10571, doi:10.1038/s41598-022-14664-2.
48. Lopez-Lazaro, M. Distribution and Biological Activities of the Flavonoid Luteolin. *Mini-Rev. Med. Chem.* **2009**, *9*, 31–59, doi:10.2174/138955709787001712.
49. Lotfi, N.; Yousefi, Z.; Golabi, M.; Khalilian, P.; Ghezelbash, B.; Montazeri, M.; Shams, M.H.; Baghbadorani, P.Z.; Eskandari, N. The Potential Anti-Cancer Effects of Quercetin on Blood, Prostate and Lung Cancers: An Update. *Front. Immunol.* **2023**, *14*.
50. Azeem, M.; Hanif, M.; Mahmood, K.; Ameer, N.; Chughtai, F.R.S.; Abid, U. An Insight into Anticancer, Antioxidant, Antimicrobial, Antidiabetic and Anti-Inflammatory Effects of Quercetin: A Review. *Polym. Bull.* **2023**, *80*, 241–262, doi:10.1007/s00289-022-04091-8.
51. Rakheja, I.; Ansari, A.H.; Ray, A.; Chandra Joshi, D.; Maiti, S. Small Molecule Quercetin Binds MALAT1 Triplex and Modulates Its Cellular Function. *Mol. Ther. Nucleic Acids* **2022**, *30*, 241–256, doi:10.1016/j.omtn.2022.09.016.
52. Primikyri, A.; Chatziathanasiadou, M.V.; Karali, E.; Kostaras, E.; Mantzaris, M.D.; Hatzimichael, E.; Shin, J.-S.; Chi, S.-W.; Briasoulis, E.; Kolettas, E.; et al. Direct Binding of Bcl-2 Family Proteins by Quercetin Triggers Its pro-Apoptotic Activity. *ACS Chem. Biol.* **2014**, *9*, 2737–2741, doi:10.1021/cb500259e.
53. Ko, C.-C.; Chen, Y.-J.; Chen, C.-T.; Liu, Y.-C.; Cheng, F.-C.; Hsu, K.-C.; Chow, L.-P. Chemical Proteomics Identifies Heterogeneous Nuclear Ribonucleoprotein (hnRNP) A1 as the Molecular Target of Quercetin in Its Anti-Cancer Effects in PC-3 Cells *. *J. Biol. Chem.* **2014**, *289*, 22078–22089, doi:10.1074/jbc.M114.553248.
54. Shin, E.J.; Lee, J.S.; Hong, S.; Lim, T.-G.; Byun, S. Quercetin Directly Targets JAK2 and PKCδ and Prevents UV-Induced Photoaging in Human Skin. *Int. J. Mol. Sci.* **2019**, *20*, 5262, doi:10.3390/ijms20215262.
55. Almatroodi, S.A.; Alsahli, M.A.; Almatroudi, A.; Verma, A.K.; Aloliqi, A.; Allemailem, K.S.; Khan, A.A.; Rahmani, A.H. Potential Therapeutic Targets of Quercetin, a Plant Flavonol, and Its Role in the Therapy of Various Types of Cancer through the Modulation of Various Cell Signaling Pathways. *Molecules* **2021**, *26*, 1315, doi:10.3390/molecules26051315.
56. Tsuchiya, A.; Kobayashi, M.; Kamatari, Y.O.; Mitsunaga, T.; Yamauchi, K. Development of Flavonoid Probes and the Binding Mode of the Target Protein and Quercetin Derivatives. *Bioorg. Med. Chem.* **2022**, *68*, 116854, doi:10.1016/j.bmc.2022.116854.
57. Gonzalez, O.; Fontanes, V.; Raychaudhuri, S.; Loo, R.; Loo, J.; Arumugaswami, V.; Sun, R.; Dasgupta, A.; French, S.W. The Heat Shock Protein Inhibitor Quercetin Attenuates Hepatitis C Virus Production. *Hepatol. Baltim. Md* **2009**, *50*, 10.1002/hep.23232, doi:10.1002/hep.23232.
58. Notas, G.; Nifli, A.-P.; Kampa, M.; Pelekanou, V.; Alexaki, V.-I.; Theodoropoulos, P.; Vercauteren, J.; Castanas, E. Quercetin Accumulates in Nuclear Structures and Triggers Specific Gene Expression in Epithelial Cells. *J. Nutr. Biochem.* **2012**, *23*, 656–666, doi:10.1016/j.jnutbio.2011.03.010.
59. Gornowicz, A.; Bielawska, A.; Szymanowski, W.; Gabryel-Porowska, H.; Czarnomysy, R.; Bielawski, K. Mechanism of Anticancer Action of Novel Berenil Complex of Platinum(II) Combined with Anti-MUC1 in MCF-7 Breast Cancer Cells. *Oncol. Lett.* **2018**, *15*, 2340–2348, doi:10.3892/ol.2017.7623.
60. Kuriakose, S.; Uzonna, J.E. Diminazene Aceturate (Berenil), a New Use for an Old Compound? *Int. Immunopharmacol.* **2014**, *21*, 342–345, doi:10.1016/j.intimp.2014.05.027.
61. Mikek, C.G.; West, S.J.; Gwin, J.C.; Dayal, N.; Sintim, H.O.; Lewis, E.A. Berenil Binds Tightly to Parallel and Mixed Parallel/Antiparallel G-Quadruplex Motifs with Varied Thermodynamic Signatures. *ACS Omega* **2018**, *3*, 11582–11591, doi:10.1021/acsomega.8b01621.

62. Reddy, B.S.P.; Sondhi, S.M.; Lown, J.W. Synthetic DNA Minor Groove-Binding Drugs☆. *Pharmacol. Ther.* **1999**, *84*, 1–111, doi:10.1016/S0163-7258(99)00021-2.
63. Pilch, D.S.; Kirolos, M.A.; Liu, X.; Plum, G.E.; Breslauer, K.J. Berenil [1,3-Bis(4′-Amidinophenyl)Triazene] Binding to DNA Duplexes and to a RNA Duplex: Evidence for Both Intercalative and Minor Groove Binding Properties. *Biochemistry* **1995**, *34*, 9962–9976, doi:10.1021/bi00031a019.
64. Pilch, D.S.; Breslauer, K.J. Ligand-Induced Formation of Nucleic Acid Triple Helices. *Proc. Natl. Acad. Sci.* **1994**, *91*, 9332–9336, doi:10.1073/pnas.91.20.9332.
65. Portugal, J. Berenil Acts as a Poison of Eukaryotic Topoisomerase II. *FEBS Lett.* **1994**, *344*, 136–138, doi:10.1016/0014-5793(94)00363-7.
66. Xi, H.; Gray, D.; Kumar, S.; Arya, D.P. Molecular Recognition of Single-Stranded RNA: Neomycin Binding to Poly(A). *FEBS Lett.* **2009**, *583*, 2269–2275, doi:10.1016/j.febslet.2009.06.007.
67. Mao, T.; Kim, J.; Peña-Hernández, M.A.; Valle, G.; Moriyama, M.; Luyten, S.; Ott, I.M.; Gomez-Calvo, M.L.; Gehlhausen, J.R.; Baker, E.; et al. Intranasal Neomycin Evokes Broad-Spectrum Antiviral Immunity in the Upper Respiratory Tract. *Proc. Natl. Acad. Sci. U. S. A.* **2024**, *121*, e2319566121, doi:10.1073/pnas.2319566121.
68. Ranjan, N.; Andreasen, K.F.; Arora, Y.; Xue, L.; Arya, D.P. Surface Dependent Dual Recognition of a G-Quadruplex DNA With Neomycin-Intercalator Conjugates. *Front. Chem.* **2020**, *8*, doi:10.3389/fchem.2020.00060.
69. Shaw, N.N.; Xi, H.; Arya, D.P. Molecular Recognition of a DNA:RNA Hybrid: Sub-Nanomolar Binding by a Neomycin–Methidium Conjugate. *Bioorg. Med. Chem. Lett.* **2008**, *18*, 4142–4145, doi:10.1016/j.bmcl.2008.05.090.
70. Wallis, M.G.; von Ahsen, U.; Schroeder, R.; Famulok, M. A Novel RNA Motif for Neomycin Recognition. *Chem. Biol.* **1995**, *2*, 543–552, doi:10.1016/1074-5521(95)90188-4.
71. Herrmann, E.; Gierschik, P.; Jakobs, K.H. Neomycin Induces High-Affinity Agonist Binding of G-Protein-Coupled Receptors. *Eur. J. Biochem.* **1989**, *185*, 677–683, doi:10.1111/j.1432-1033.1989.tb15165.x.
72. Gabev, E.; Kasianowicz, J.; Abbott, T.; McLaughlin, S. Binding of Neomycin to Phosphatidylinositol 4,5-Bisphosphate (PIP2). *Biochim. Biophys. Acta* **1989**, *979*, 105–112, doi:10.1016/0005-2736(89)90529-4.
73. Faber, C.; Sticht, H.; Schweimer, K.; Rösch, P. Structural Rearrangements of HIV-1 Tat-Responsive RNA upon Binding of Neomycin B*. *J. Biol. Chem.* **2000**, *275*, 20660–20666, doi:10.1074/jbc.M000920200.
74. Wang, S.; Huber, P.W.; Cui, M.; Czarnik, A.W.; Mei, H.-Y. Binding of Neomycin to the TAR Element of HIV-1 RNA Induces Dissociation of Tat Protein by an Allosteric Mechanism. *Biochemistry* **1998**, *37*, 5549–5557, doi:10.1021/bi972808a.
75. McFarland Jr., A.W.; Fernando, L.P.; Kellish, P.; Story, S.P.; Schober, G.B.; Kumar, S.; Gong, C.; King, A.; Gong, X.; Leutou, A.S.; et al. Nucleic Acid Specificity, Cellular Localization and Reduced Toxicities of Thiazole Orange-Neomycin Conjugates. *ChemistryOpen* **2025**, *14*, e202400189, doi:10.1002/open.202400189.
76. Nakagawa, S.; Ip, J.Y.; Shioi, G.; Tripathi, V.; Zong, X.; Hirose, T.; Prasanth, K.V. Malat1 Is Not an Essential Component of Nuclear Speckles in Mice. *RNA* **2012**, *18*, 1487–1499, doi:10.1261/rna.033217.112.
77. Zhang, N.; Lan, R.; Chen, Y.; Hu, J. Identification of KDM4C as a Gene Conferring Drug Resistance in Multiple Myeloma. *Open Life Sci.* **2024**, *19*, 20220848, doi:10.1515/biol-2022-0848.
78. Tripathi, V.; Ellis, J.D.; Shen, Z.; Song, D.Y.; Pan, Q.; Watt, A.T.; Freier, S.M.; Bennett, C.F.; Sharma, A.; Bubulya, P.A.; et al. The Nuclear-Retained Noncoding RNA MALAT1 Regulates Alternative Splicing by Modulating SR Splicing Factor Phosphorylation. *Mol. Cell* **2010**, *39*, 925–938, doi:10.1016/j.molcel.2010.08.011.

79. Wang, F.; Li, Y.; Zhou, J.; Xu, J.; Peng, C.; Ye, F.; Shen, Y.; Lu, W.; Wan, X.; Xie, X. miR-375 Is Down-Regulated in Squamous Cervical Cancer and Inhibits Cell Migration and Invasion via Targeting Transcription Factor SP1. *Am. J. Pathol.* **2011**, *179*, 2580–2588, doi:10.1016/j.ajpath.2011.07.037.
80. Brown, J.A.; Kinzig, C.G.; DeGregorio, S.J.; Steitz, J.A. Methyltransferase-like Protein 16 Binds the 3′-Terminal Triple Helix of MALAT1 Long Noncoding RNA. *Proc. Natl. Acad. Sci.* **2016**, *113*, 14013–14018, doi:10.1073/pnas.1614759113.
81. Dumbović, G.; Braunschweig, U.; Langner, H.K.; Smallegan, M.; Biayna, J.; Hass, E.P.; Jastrzebska, K.; Blencowe, B.; Cech, T.R.; Caruthers, M.H.; et al. Nuclear Compartmentalization of TERT mRNA and TUG1 lncRNA Is Driven by Intron Retention. *Nat. Commun.* **2021**, *12*, 3308, doi:10.1038/s41467-021-23221-w.
82. Yang, W.; Soares, J.; Greninger, P.; Edelman, E.J.; Lightfoot, H.; Forbes, S.; Bindal, N.; Beare, D.; Smith, J.A.; Thompson, I.R.; et al. Genomics of Drug Sensitivity in Cancer (GDSC): A Resource for Therapeutic Biomarker Discovery in Cancer Cells. *Nucleic Acids Res.* **2013**, *41*, D955-961, doi:10.1093/nar/gks1111.
83. Arun, G.; Aggarwal, D.; Spector, D.L. MALAT1 Long Non-Coding RNA: Functional Implications. *Non-Coding RNA* **2020**, *6*, 22, doi:10.3390/ncrna6020022.
84. Kwok, Z.H.; Roche, V.; Chew, X.H.; Fadieieva, A.; Tay, Y. A Non-Canonical Tumor Suppressive Role for the Long Non-Coding RNA MALAT1 in Colon and Breast Cancers. *Int. J. Cancer* **2018**, *143*, 668–678, doi:10.1002/ijc.31386.
85. Sasaki, Y.T.F.; Ideue, T.; Sano, M.; Mituyama, T.; Hirose, T. MENε/β Noncoding RNAs Are Essential for Structural Integrity of Nuclear Paraspeckles. *Proc. Natl. Acad. Sci.* **2009**, *106*, 2525–2530, doi:10.1073/pnas.0807899106.
86. Shen, X.; Ye, Z.; Wu, W.; Zhao, K.; Cheng, G.; Xu, L.; Gan, L.; Wu, Y.; Yang, Z. lncRNA NEAT1 Facilitates the Progression of Colorectal Cancer via the KDM5A/Cul4A and Wnt Signaling Pathway. *Int. J. Oncol.* **2021**, *59*, 1–12, doi:10.3892/ijo.2021.5231.
87. Li, Y.; Xu, Y.; Yu, X.; Su, S.; Wu, B.; Su, Y.; Guo, L. Long Non-Coding RNA NEAT1 Promotes Colorectal Cancer Progression via Interacting with SIRT1. *Sci. Rep.* **2025**, *15*, 5673, doi:10.1038/s41598-025-90416-2.
88. Dumbović, G.; Braunschweig, U.; Langner, H.K.; Smallegan, M.; Biayna, J.; Hass, E.P.; Jastrzebska, K.; Blencowe, B.; Cech, T.R.; Caruthers, M.H.; et al. Nuclear Compartmentalization of TERT mRNA and TUG1 lncRNA Is Driven by Intron Retention. *Nat. Commun.* **2021**, *12*, 3308, doi:10.1038/s41467-021-23221-w.
